# Gold Nanorods for LSPR Biosensing: Synthesis, Coating by Silica, and Bioanalytical Applications

**DOI:** 10.3390/bios10100146

**Published:** 2020-10-17

**Authors:** Vincent Pellas, David Hu, Yacine Mazouzi, Yoan Mimoun, Juliette Blanchard, Clément Guibert, Michèle Salmain, Souhir Boujday

**Affiliations:** 1 Laboratoire de Réactivité de Surface (LRS), Sorbonne Université, CNRS, UMR 7197, 4 Place Jussieu, F-75005 Paris, France; vincent.pellas@sorbonne-universite.fr (V.P.); david.hu@sorbonne-universite.fr (D.H.); yacine.mazouzi@sorbonne-universite.fr (Y.M.); yoan.mimoun@etu.sorbonne-universite.fr (Y.M.); juliette.blanchard@sorbonne-universite.fr (J.B.); clement.guibert@sorbonne-universite.fr (C.G.); 2 Institut Parisien de Chimie Moléculaire (IPCM), Sorbonne Université, CNRS, 4 Place Jussieu, F-75005 Paris, France

**Keywords:** gold nanorods, silica coating, localized surface plasmon resonance (LSPR), surface functionalization

## Abstract

Nanoparticles made of coinage metals are well known to display unique optical properties stemming from the localized surface plasmon resonance (LSPR) phenomenon, allowing their use as transducers in various biosensing configurations. While most of the reports initially dealt with spherical gold nanoparticles owing to their ease of synthesis, the interest in gold nanorods (AuNR) as plasmonic biosensors is rising steadily. These anisotropic nanoparticles exhibit, on top of the LSPR band in the blue range common with spherical nanoparticles, a longitudinal LSPR band, in all respects superior, and in particular in terms of sensitivity to the surrounding media and LSPR-biosensing. However, AuNRs synthesis and their further functionalization are less straightforward and require thorough processing. In this paper, we intend to give an up-to-date overview of gold nanorods in LSPR biosensing, starting from a critical review of the recent findings on AuNR synthesis and the main challenges related to it. We further highlight the various strategies set up to coat AuNR with a silica shell of controlled thickness and porosity compatible with LSPR-biosensing. Then, we provide a survey of the methods employed to attach various bioreceptors to AuNR. Finally, the most representative examples of AuNR-based LSPR biosensors are reviewed with a focus put on their analytical performances.

## 1. Introduction

The part taken by plasmonic nanoparticles in biotechnologies has been expanding steadily over the last few decades. The popularity of these nano-objects stems from the localized surface plasmon resonance (LSPR) phenomenon that leads to an intense absorbance band at certain resonance frequencies [1,2]. This outstanding property is at the origin of their implementation in multiple biomedical applications including biosensing [3,4], therapy, and theranosis [5,6]. Among the multiple features of plasmonic nanoparticles, an interesting property, namely the extreme sensitivity of the LSPR band to minor changes of the dielectric constant/refractive index (RI) of the local environment, gave rise to the LSPR-based biosensing [7]. This sensitivity allows for the detection of binding events through standard absorption spectroscopy measurements or even by visual detection [8,9,10,11,12].

Although the biomedical story originated with spherical gold nanoparticles, anisotropic particles, and in particular gold nanorods (AuNRs), are increasingly dominating the field. Indeed, while spherical gold nanoparticles exhibit a unique LSPR band around 520 nm, the two dimensions of AuNRs lead to two absorption bands, the first, also located around 520 nm, is due to the transverse localized surface plasmon resonance (t-LSPR), and the second, far more advantageous, is due to the longitudinal localized surface plasmon resonance (l-LSPR). The l-LSPR band is located at higher wavelengths, matching the first (650–950 nm) or the second (1000–1350 nm) biological window [13]. The position of the l-LSPR band can be tuned by modulating the aspect ratio (AR) of AuNRs rather than their length [14]. As a result, AuNRs have become the ideal candidates for a wide range of biomedical applications [13,15,16,17,18].

In the biosensing field, along with the other biomedical applications, the use of AuNRs is also expanding significantly and AuNRs are proving to be versatile and multipurpose actors allowing for the read-out of the transduction through multiple scenarios. For instance, they can be used to enhance the response of existing transducing techniques such as propagative SPR [19,20,21] or to quench and/or enhance the fluorescence signal [22,23]. They are also capable of achieving the transduction through an etching/growth balance [24]. Finally, they are extremely efficient candidates for LSPR-biosensing, the application on which we concentrate in this review and which, until quite recently, had been dominated by spherical nanoparticles. This boom is mainly the consequence of the fact that AuNRs’ l-LSPR displays a higher sensitivity to variation of the local dielectric environmental compared to the LSPR band of spherical nanoparticles [25]. Nevertheless, despite their attractiveness, AuNRs have a significant number of disadvantages that limit their use on a larger scale as LSPR-biosensors. In particular, their synthesis seems to be subject to multiple approximations leading to difficulties in reproducing the ARs as well as a limitation of upscaling. In addition, the indispensable use of stabilizing agents strongly bound to the AuNRs’ surface, such as CTAB, considerably complicates their surface chemistry and, by implication, the grafting of receptors necessary for the operation of the LSPR-biosensors. AuNRs coating with silica partially solves the second problem by conferring them a biocompatibility and a more practicable surface chemistry, however it also suffers from lack of reproducibility of their synthesis, which makes difficult the control of the thickness and, more generally, the quality of the silica shell.

In what follows, we review the recent progresses related to AuNRs for LSPR biosensing starting from their synthesis and coating by silica, then proceeding to their characterization and further surface functionalization, and ending with a selection of bioanalytical applications. In the past few decades, much progress has been made in the mastering of AuNRs synthesis and the underlying mechanism as summarized in these reviews dated in 2009 and 2013 [17,26]. In the first part of the section, we build on these reviews and discuss the more recent literature on the subject, with a special focus on the problems that may interfere with the subsequent use of these objects for biosensing. The second part of the synthesis section is devoted to silica coating although very little examples of LSPR biosensing using AuNR@SiO_2_ can be found in the literature. This poor use of such promising objects is mainly driven by the difficulty to achieve thin layers of silica on top of the nanorods. When the silica shell is thick, the LSPR signal at its surface decays and, as a consequence, no change is recorded upon target recognition. However, herein, we intend to demonstrate that the silica coating of AuNRs has reached a level of maturity allowing for the precise tuning of the thickness of the silica layer down to the nanometer level and even below, which paves the way for their extensive use in LSPR biosensing. In the second section of this manuscript, we review the characterization methods allowing for the investigation of the structure and shape of gold nanorods and list, when possible, the means for the characterization of the outer silica shell. The third section is devoted to AuNRs surface functionalization, mandatory to attach the biological element responsible for target recognition prior to their use for LSPR-biosensing. We review in this part the strategies together with the relevant molecules allowing for AuNRs surface functionalization by physisorption, chemisorption, or conjugation. The last part of this manuscript is devoted to the applications of AuNRs in LSPR biosensing, i.e., the biosensors for which read-out is based on a shift in the position and/or intensity of the LSPR band. The main bioreceptors associated with AuNRs are antibodies and aptamers used either in solution or in solid phase. We recap the different strategies and discuss the analytical performances, i.e., the limit of detection (LoD) and the detection range (DR) put in perspective with other transduction techniques. We finally discuss the use of AuNRs as single molecules plasmonic biosensors.

## 2. Synthesis Methods

**Figure 1:**
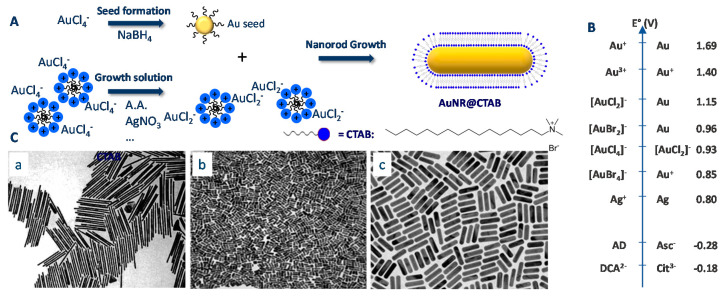
(**A**) Synthesis of gold nanorods (AuNRs) from crystal seed according to the seed-mediated growth method, and (**B**) standard potential in aqueous solution of different Red/Ox couples playing a role in the reaction of AuNR formation [32,33,34] and (**C**) TEM images of AuNRs synthesized following (**a**) Jana et al.’s method [28], (**b**) Sau et al.’s method [29], and (**c**) Ye et al.’s method [30].

Most AuNR syntheses rely on the so-called seed-growth method as shown in Figure 1A. The seed-mediated growth process involves two separated steps: First, the synthesis of tiny spherical seed nanoparticles and second, their subsequent growth in a solution containing metal precursors, weak reducing agent such as ascorbic acid, and a capping agent, usually CTAB (hexadecyltrimethylammonium bromide), a cationic surfactant. CTAB adsorbs on AuNRs surface, and allows both their directional growth and electrostatic stabilization through the formation of a dense double layer [27]. The synthesis protocol is regulated by the balance of redox potentials (redox potentials of the main couples involved in AuNR synthesis are reported in Figure 1B). The TEM images shown in Figure 1C are extracted from the work of Jana et al., Sau et al., and Ye et al. [28,29,30], respectively, to highlight how the synthesis protocol has reached a level of maturity allowing for a quasi-perfect homogeneity of the rods. For detailed mechanistic investigation in AuNRs formation and parameters influencing their growth, we refer to the review published by El-Sayed et al. in 2009 [17] and Murphy et al. in 2013 [26,31]. In what follows, we will briefly introduce a historical background of AuNR synthesis, discuss the origins in the lack of reproducibility, difficulties encountered during the synthesis, storage issues, and finally, the complexity of scaling up the synthesis.

### 2.1. Gold Nanorods

#### 2.1.1. Historical Background

The very first reported method for the synthesis of AuNR, which dates back to 1994, was based on the electrochemical reduction of a gold precursor (HAuCl_4_) within a porous membrane. However, this method had many limitations, among which was a very low yield [35]. The breakthrough in synthesis and applications of AuNRs really began at the dawn of the 21st century, when Jana et al. reported the synthesis of AuNRs by a seeding growth method [36]. In their initial protocol, a solution of 3.5 nm citrate-stabilized Au seeds is injected into a growth solution containing HAuCl_4_, cetyltrimethylammonium bromide (CTAB), L-ascorbic acid (AA), and silver nitrate; the AR was varied by changing the amount of seed solution introduced in the growth solution. Shortly after this first publication, Jana et al. reported on the possibility to synthesize AuNRs with high AR (as high as 18, dimensions 400 × 25 nm) by performing successive (up to three) growth steps, in three identical growth solutions [28]. Later, in 2003, Nikoobakht et al. introduced the use of a seed solution containing smaller (ca. 1.5 nm), CTAB-stabilized, Au seeds and a growth solution containing a binary mixture of surfactants (benzyldodecylammonium chloride (BDAC) + CTAB), silver nitrate, and AA. This second synthesis protocol produces thinner AuNR (diameter ca. 12 nm) and with ARs in the range 1.5–4.5 [37]. The yield in NR (compared to other shapes of AuNPs) was relatively low in this initial report, and in the method developed by Jana et al., but Murray et al. significantly improved the yield toward AuNRs (~97%) of this second synthesis protocol for low AR (2–4) AuNRs [29]. Another protocol was developed shortly after that, which is often qualified as seedless, although it is rather a one-step seeded protocol. This third protocol, initially developed by Jana et al. [38], is actually a modification of the second one, where the seeds are grown in situ, in the growth solution, by the rapid addition of NaBH_4_, a strong reducing agent. The use of this strong reducing agent allows for separating the nucleation step (fast reduction with NaBH_4_) from the growth step (slower reduction with AA). Moreover, by using a growth solution containing a high concentration of gold, it allows the gram-scale production of AuNR. The AuNRs obtained with this protocol have significantly smaller dimensions (with diameter as low as 2.5 nm). This “seedless” protocol initially suffered from a large distribution in ARs and the presence of a high fraction of nanospheres (that were difficult to separate from these small AuNRs), but further developments and optimization allowed the synthesis of AuNRs with well controlled ARs and negligible contamination by nanospheres [39]. It is important to mention here that these small nanorods have lower extinction coefficients (for an l-LSPR band at 800 nm: ca. 26 times lower per AuNR and ca. 2.5 times lower per gold atom i.e., with normalization by the volume of the NRs) than the larger nanorods produced by the first and second methods, because they scatter light less strongly [39]. These lower extinction coefficients may not be favorable for their application as LSPR biosensors.

The synthesis of AuNRs has been the subject of two comprehensive reviews, first in 2009 by El-Sayed et al. [17] and then in 2013 by Murphy et al. [26]. At that time, the optimization of the protocols for the three synthetic routes was already well under way, including the identification of the main parameters allowing to control the AR of the AuNRs. We will focus this section dedicated to the synthesis of AuNRs on the new developments since 2013, based on the second synthesis protocol (CTAB-stabilized seeds/seeded growth) as (i) there has been very few new developments in the first synthesis protocol (citrate-stabilized seed/seeded growth) and (ii) the third protocol (“seedless” synthesis) is, as mentioned above, very similar to the second one except that the seeds are generated in situ, in the growth solution. We will concentrate on the practical aspects of the synthesis and highlight the main difficulties (mostly leaving aside the literature related to the mechanisms of formation of AuNRs discussed extensively in the above-mentioned reviews).

#### 2.1.2. Origins of the Lack of Reproducibility in the Synthesis of AuNRs

**Figure 2:**
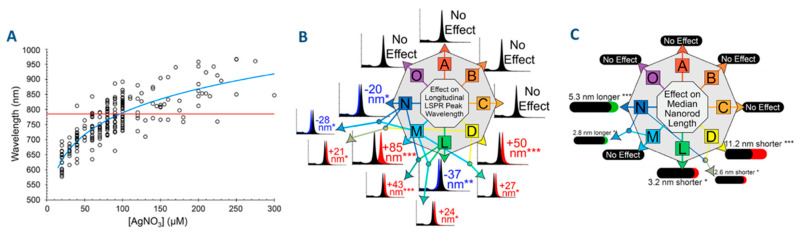
(**A**) Plot of the longitudinal surface plasmon resonance extinction peak wavelength as a function of silver nitrate concentration illustrating the synthesis variation by different individuals produced in the Murphy group over the last half-decade. (**B**) and (**C**) Graphical summary of the significant primary and secondary interaction effects on the l- localized surface plasmon resonance (LSPR) peak wavelength and of the median nanorod length: Amount of NaBH_4_ (A), stirring rate of the seed solution (B), age of seed solution (C), amount of seed (D), temperature (L), amount of silver (M), amount of ascorbic acid (N), and age of reduced solution (O). P values (***) < 0.001 < (**) < 0.01 < (*) < 0.05 (from variance analysis). Adapted from [40].

Synthesis of AuNRs with different ARs (and, as a consequence different positions of their l-LSPR bands) does not require advanced synthetic skills or very complicated set-ups. For example, a shift of the position of the l-LSPR band over 300 nm can be obtained by changing the concentration of silver nitrate in the growth solution. Another simple way to alter the position of the l-LSPR band is to change the amount of added Au seeds. Nevertheless, one cannot deny that difficulties are often encountered when performing these syntheses. The main issue is certainly, as emphasized by Burrows et al., the lack of reproducibility [40]. While, for an (experienced) individual, it is possible to continuously adjust the position of the l-LSPR band from 600 to 1000 nm by gradually increasing the concentration of AgNO_3_, for experiments performed by several individuals, variation of the l-LSPR band of about 150 nm can be observed for apparently identical synthesis conditions (Figure 2A). This is a major problem when the purpose is to produce AuNRs with a given l-LSPR position: The horizontal red line on Figure 2A shows that AuNRs with l-LSPR at a given position (785 nm, which corresponds to a typical laser wavelength used in biomedical applications) have been obtained for AgNO_3_ concentration ranging between 60 and 220 μmol. This issue is actually to be expected considering the many components and steps that enter in the synthesis of AuNRs.

As mentioned by Scarabelli et al. in their practical guide, it is of paramount importance to avoid sources of poor reproducibility such as a low purity of the CTAB source (a too high amount of iodide leads to a low shape selectivity toward AuNRs [41]) or of water, insufficient cleaning of glassware, inappropriate, or too long storage of the stock solutions [42]. These authors also emphasized that the addition of NaBH_4_ to the AuCl_4_^-^/CTAB solution should be performed very fast in order to trigger a burst of nucleation and obtain small Au seeds with a good size homogeneity.

Regarding the possible sources of variations in the AR of AuNRs, Scarabelli et al. identified, based on an analysis of the literature that increasing the Ag^+^ concentration or amount of Au seeds, or decreasing the pH or the AA concentration, leads to an increase in the AR of AuNRs [42]. Reza Hormozi-Nezhad et al. have identified, using experimental plans, that the position of the l-LSPR band can also be shifted by increasing CTAB concentration, but to a lower extent [43]. Also using experimental design, Burrows et al. [40] have identified the temperature of synthesis as another key parameter: The l-LSPR band of AuNRs blue-shifts upon increasing the temperature of reaction as shown Figure 2B (this last parameter is however limited by the fact that the properties of CTAB, and especially its solubility, strongly depend on the temperature). Burrows et al. also identified four synthesis parameters that had no influence on the formation of AuNRs: Amount of NaBH_4_ used for the preparation of the seed solution; rate of stirring and age of seed solution; and aging of reduced growth solution prior to the addition of seeds. Moreover, they investigated the influence of these synthesis parameters on other aspects of the synthesis such as the yield in gold, the fraction of AuNRs (vs. particles of other shape) and the width and length of the AuNRs. Some of these results are, however, questionable: For example, the fact that the concentration of silver strongly affects the position of the l-LSPR band (expected result) while it does not modify the length and width of AuNRs (and hence, the AR, Figure 2B,C), a conclusion that is really unexpected based on previous literature; the absence of effect of seed aging is also at variance with previous works, especially the work of Watt et al., which clearly established, based on SAXS experiments, an aging of the seeds through Oswald Ripening within a few hours and its detrimental impact on the formation of AuNRs [44]; the absence of detrimental impact of an increase of the concentration of AA on the selectivity toward the formation of AuNRs is also at variance with previous works [29,45], but this is due to the fact that the authors deliberately choose to work with an sub-stoichiometric amount of AA (with respect to Au). It is important to mention here that Hormozi-Nezhad et al. [43] and Burrows et al. [40] both concluded that the identified parameters did not simply act additively but also, for some of them, synergistically. The existence of secondary interactions between some of the synthesis parameters indicates that these parameters are mechanistically connected.

Another important conclusion of the work of Burrows et al. is that the variations in the position of l-LSPR band that are observed during their series of experiments cannot fully explain the large discrepancy (ca. 150 nm), for apparently identical experimental conditions in the position of the l-LSPR band for samples prepared by different experimenters, and that further work is needed to explore other, less obvious, parameters.

#### 2.1.3. Other Difficulties Often Encountered in the Preparation of AuNRs

In addition to the lack of reproducibility in the position of the l-LSPR band, the other aspects of the synthesis of AuNRs that hamper their industrial development are:

- Low yield in reduced gold

It is important to mention here that many authors use the term yield to describe the fraction of AuNRs (vs. AuNPs of other shapes). Hence, in many publications, “high yield” means high selectivity toward AuNRs. This can be all the more misleading as yield in gold is often not reported [29,38]. Using AA as reducer, Au is only partially reduced, leading usually to yield in reduced gold of about 15–20% [40,46]. Indeed, during the growth phase, it is important to avoid the nucleation of new Au seeds and the growth of existing Au seeds into isotropic Au NPs. Hence, the rate of reduction should be kept slow, and with AA, this can only be achieved by keeping the concentration of AA close to stoichiometry ([AA]/[AuCl_4_^-^] ≈ 1.1), while higher concentrations of AA leads to large amounts of spherical particles [45]. Several authors have proposed to replace AA by other, milder reductants such as hydroquinone (used by Vigderman et al. in 10× excess with regards to HAuCl_4_, yield in gold close to 100% based on ICP [45]), H_2_O_2_-NaOH (used by Xu et al. with an up to 300× excess with respect to HAuCl_4_, yield in reduced gold is not reported [47]), hydroxylamine (used by Leng et al. in 20× excess, yield in gold is not reported, [48] or 3-amino-phenol (used by Wu et al. in 10x stoichiometric excess, yield in gold close to 100%).

Other approaches have been proposed to increase the yield in reduced gold (while keeping a high selectivity toward AuNRs): for example Canonico-May et al. have proposed to recycle the growth solution, that is, after the separation of the AuNR from the supernatant by centrifugation, AA is added to the supernatant and the obtained solution is seeded again with Au seed and used to grow a second series of AuNR. This can be repeated up to 5 times allowing to use about 75% of the HAuCl_4_ (compared to about 17% during the first growth step based on ICP-MS measurements), while producing AuNRs with similar ARs and dimensions [49]. Similarly, Kozek et al. also increased the fraction of reduced gold by slowly adding (with a syringe pump), to the first growing solution and after a first conventional growth step, an AA aqueous solution. The yield in gold is not calculated but it is assumed to reach 100%, based on the fact that addition of more AA does not lead to a further growth of the AuNRs [50]. Chang and Murphy were able to significantly increase the fraction of reduced gold (up to 89%), while keeping the fraction of AuNR high (>95%) using AA as reducer in a 1.6 molar excess by decreasing the pH (which has the consequence to decrease the reducing power of AA). This lower pH however also leads to the formation AuNRs with smaller dimensions (diameter below 9 nm) and with relatively small AR (below 4) [51].

- Presence of Au particles with other shapes and/or too large distribution of AR.

**Figure 3:**
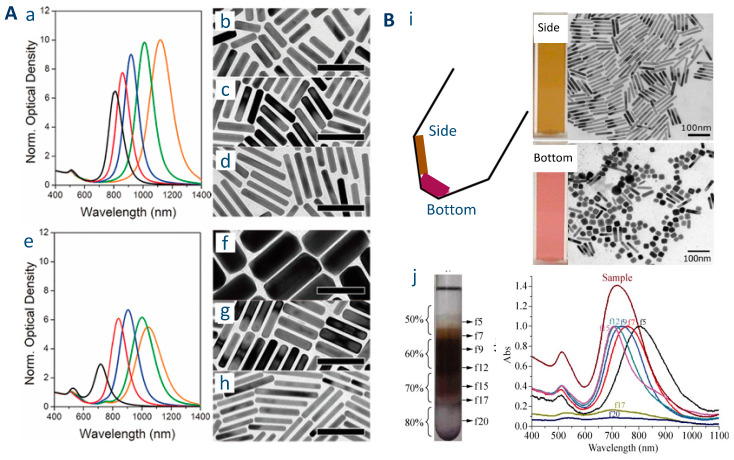
(**A**) Increasing AuNRs monodispersity: (**a**–**d**) Comparison between the use of small AuNRs seeds in the two step growth method and (**e**–**h**) standard Au nanocrystal seeds of 1–2 nm in conventional method in the growth of AuNRs at increasing [HCl]/[HAuCl_4_] ratios and their respective TEM images and normalized absorption spectra. Adapted from [53]. Scale bars: 100 nm. Purification methods: (**B**) (**i**) Separation of AuNRs from Au nanospheres after conventional centrifugation; TEM pictures of the particles deposited (top) on the side wall and (bottom) at the bottom of the centrifugation tube. Adapted from [54] and (**j**) picture of AuNR suspension after gradient centrifugation in aqueous cetyltrimethylammonium bromide (CTAB)-ethylene glycol solution and UV-vis spectra of the colloidal suspensions recovered at different positions in the centrifugation tube. Adapted from [55].

The most obvious solution to this difficulty is to use protocols with a high selectivity toward nanorods and with a narrow distribution in their dimensions. Among the many attempts at optimising the synthesis conditions in order to produce AuNRs with high shape selectivity and a narrow distribution in AR, one can cite the recent and very promising work of González-Rubio et al.: one origin of polydispersity in AuNR dimensions is the fact that symmetry breaking (from cuboctahedral NPs to nanorods via the emergence of (110) and (250) facets) occurs asynchronously during the growth step. González-Rubio et al. have developed a protocol including two growth steps: the Au seeds are first fully transformed to very small AuNRs (first growth step) and these small AuNR are further grown to larger AuNR (second growth step) [52,53]. By doing so, they dissociate the symmetry breaking step (which occurs during the first growth step) from the anisotropic growth step (second growth step). The key step in this protocol is the formation of small AuNR thanks to the addition of n-decanol, a cosurfactant. This protocol leads to AuNRs with a narrow l-LSPR band whose position can be tuned over a wide spectral range (from 600 to 1270 nm). The gain in absorbance, at similar wavelength between the colloidal suspensions of AuNRs prepared with this method compared to the conventional two-step method, is close to a factor 2 (Figure 3A).

Post-synthesis removal of the small fraction of AuNRs of other shapes, or separation of AuNRs as a function of their size can also be used to increase sensitivity. Purification of AuNR can be performed by several methods. The very small AuNPs (<5 nm) will usually remain in the supernatant. The easiest method to separate “large” spherical particles from AuNRs has been proposed by Sharma et al. They observed that, after centrifugation, two spots of different colors could be observed in the centrifugation tube. The analysis by TEM and UV-vis reveals that the part of the sample located in the walls is highly enriched in NRs, while the part of the sample located at the bottom of the centrifugation tube contains mostly isotropic AuNPs [54] (Figure 3B). This protocol of separation by conventional centrifugation was further refined by Boksebeld et al. who introduced a succession of three short (1 min) centrifugation steps at 6700 g, in order to selectively precipitate gold nanospheres while leaving gold nanorod in the supernatant. This protocol was successfully applied to rather “short” (AR 2.4, 3.7 and 5.3) AuNRs [56]. More complex separation can be performed using depletion-induced aggregation [57]. This method is very efficient but requires the addition of large amounts of CTAB and therefore increases further the total cost of the synthesis (see next section). A fine purification of AuNRs can also be achieved using density gradient centrifugation: In this protocol, a centrifugation tube is filled with layers of solutions of different densities prepared by mixing ethylene glycol with aqueous CTAB solution (50–80% of EG by volume), and the raw suspension is deposited on the top of these layers. After centrifugation, the AuNPs will remain in different layers depending on their dimensions [55] (Figure 3C).

- High cost of synthesis

When preparing gold nanoparticles, one would certainly expect the main source of cost to be the gold precursor. However, as emphasized by Xu et al., due to the high concentration of surfactant required for AuNRs synthesis and to the necessity of using high purity surfactant, the estimated cost of surfactant is 85% of the total cost of production of AuNRs in the initial synthesis process (pure CTAB as surfactant, Nikoobakht and El-Sayed protocol [37]) and decreases to 50% when a mixture of CTAB and NaOL is used, following the protocol of Ye et al. [30]). In their estimation of the contribution of the surfactant to the total cost of the synthesis, Uson et al. calculated a slightly lower contribution for surfactant (ca. 58%) for a synthesis protocol using only CTAB [58]. These two calculations indicate that, in order to achieve industrial scale production of AuNRs, the contribution of surfactant to the total cost must be reduced. To this purpose, Xu et al. have successfully replaced CTAB by a gemini surfactant (maleic acid diethyl bis(hexadecyl dimethylammonium bromide (P16-8-16)) of industrial grade and with a much lower critical micellar concentration (CMC). With this surfactant, the gold precursor becomes by far the major source of cost (99.3%). Moreover, this surfactant participates in the reduction of the gold precursor thanks to its C = C double bond [59]. Reaching a high yield in reduced gold is, of course, also one of the keys to decrease the cost of synthesis, but this aspect has already been discussed in this review. It is also important to remind here that the major cost may come from the post-modification of AuNRs: For example, Uson et al. have estimated that a PEGylation step can account for 2/3 of the cost [58].

#### 2.1.4. Short Shelf-Life of AuNRs/Poor Stability of AuNRs in Oxidizing Conditions

Storage conditions play an important role in the shelf-life of AuNRs. Kaur and Chudasama have confirmed that AuNRs should not be stored in their growth solution, because they continue to age and this leads to a progressive blue-shift of their l-LSPR band; storage in a CTAB-rich solution is not desirable either as it also leads to a progressive blue-shift (although slower and less pronounced) of the l-LSPR band; on the other side, dispersion of AuNRs in water (leading, in fact, to a CTAB-poor solution) allows preserving the properties of AuNRs over a period of 30 days [60].

One potential limitation of the application of AuNRs as sensors is their instability in an oxidative environment. This instability results in a progressive blue-shift of the l-LSPR band associated with a broadening of this band and a decrease of intensity. Ultimately, complete dissolution of AuNR to AuBr_4_^-^ occurs. Vassilini et al. have observed that the oxidative dissolution of AuNR by H_2_O_2_ is strongly slowed down for AuNR with larger diameter. They also observed that reducing the amount of Br^-^ is key to produce oxidant resistant AuNR and explain the negative impact of Br^-^ on the stability of AuNR by a lowering of the oxidation potential of gold in presence of this anion. They demonstrate that, thanks to this improve in stability, AuNRs can be used as SERS substrate for monitoring the oxidation of crystal violet (CV) and that the AuNRs can be recycled (up to 10×) without modification in the intensity of the SERS signal of adsorbed CV [61].

#### 2.1.5. Difficulties in Scaling up the Synthesis

The amount of AuNRs produced during a conventional synthesis is fairly small: ca. 7 mg from 100 mL of aqueous solution [62]. This is due to the fact that this synthesis is performed in very dilute solutions and that the yield in reduced gold is often relatively low. Hence, syntheses that are described as “large-scale” often produce ca. 100–200 mg of AuNRs for 1 L of aqueous solution [50]. The very first report of gram-scale synthesis of AuNRs was by Jana et al. in 2005 (using a “seedless” synthesis protocol) but this gram-scale production was obtained at the expense of a loss of control over product properties (higher polydispersity in the dimension of the nanorods) [38]. Scaling-up of the synthesis of AuNRs could, in principle, be achieved by two different approaches: Either by a volumetric scale up (that is, by keeping all concentrations identical to the standard protocol) or by increasing the concentrations of reactants in the solution.

**Figure 4:**
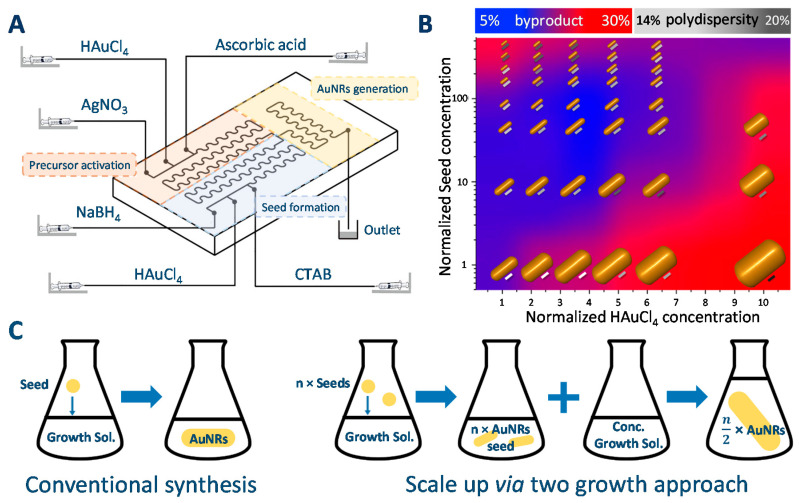
(**A**) Microfluidic flow device allowing better control of reaction parameters and reduction of AuNRs polydispersity. Adapted from [63]. (**B**) Impact of increasing seed and reactant concentration on the structural characteristics of the obtained AuNRs. AuNRs obtained by the conventional seed-mediated protocol is represented as 1/1 ratio in seed/HAuCl_4_ normalized concentration (polydispersity of AR is reflected by the color of the bar next to the AuNR and product purity by the shade of background color) Adapted from [64]. (**C**) Conventional one-step growth approach (left) compared to the two-step growth approach developed by Park et al. (right) with increasing seeds and second growth solution concentration Adapted from [64].

An obvious limitation of the first of these two approaches (that is, increasing the volume of the solution) is that the subsequent post-processing steps (centrifugation and washing) will require handling large volumes of solution. Moreover, an attempt to increase the volume of reaction is hampered by the fact that AuNRs produced from larger volumes usually have a higher polydispersity in their dimensions, probably because higher volumes alter rates of thermal transport and reagent diffusion [62]. Successful scaling up of the synthesis to 1 L solution has been reported by Kozek et al. [50] and Chang and Murphy [51] for batch synthesis, while Lohse et al. reported a production rate of ca. 0.5 mg/min using a millifluidic set-up [62]. Synthesis of AuNRs using microfluidic reactors (either using a chip reactor [44,63] or micrometric tubings [58]) have also been reported. However, as mentioned by Lohse et al. reaching gram-scale with a microfluidic reactor would require numbering up a large number of reactors [62]. Beside scaling-up, using micro- or milli-fluidic reactors has two added advantages. Flow synthesis usually leads to a lower polydispersity in the dimensions of the nanorods [62] and it is possible to integrate all the steps of the synthesis in the same flow device (Figure 4A), that is the preparation of the seeds and growth solution [63] or post-synthesis modifications such as PEGylation [58], hence decreasing the number of synthesis steps. In the example of postsynthesis PEGylation, the cost of the synthesis is reduced by a factor of 100 in proportion of the amount of PEG-SH used. Integration of the preparation of growth and seeds solutions in the flow device probably participates in the reduced polydispersity and higher reproducibility of the AuNRs because of the continuous use of freshly prepared solutions [44].

Increasing the concentration of reactants is even more challenging as a simple increase of all concentrations results in AuNRs with high polydispersity in their dimensions and to the formation of spherical AuNPs. Park et al. have examined this approach by analyzing the influences of increasing the concentration of gold precursor in the growth solution and the amount of seeds on the quality (extent of polydispersity) and purity (extent of formation of spherical nanoparticles) for the batch synthesis of AuNRs. Their analysis confirmed that the concentration of gold in the growth solution could only be marginally increased (ca. by a factor of 3) and that the concentration of Au in the growth solution and the amount of seeds should be both increased to keep a reasonably high purity (see Figure 4B). Park et al. assigned the observed decrease in quality and purity of AuNRs upon increasing the concentration of gold precursor in the growth solution to the disruption of the balance between reactant reduction and micelle adsorption on very small Au nanoparticles that is at the origin of symmetry breaking (from spherical- to nanorod-shaped NPs). Hence, they propose a two-step growth protocol (quite similar to the protocol proposed by González-Rubio et al. and described above [53]) that allows separating the symmetry breaking step (growth of isotropic AuNP into AuNRs seeds) to the growth of these anisotropic seeds (see Figure 4B). The scaling-up of the synthesis was possible up to a 200× increase in the amount of seeds and concentration of gold precursor (corresponding to a 100× higher production of AuNRs) with only minor changes in the dimensions of the nanorods [64].

One can also mention here the work of Khanal and Zubarev who reported the successful gram-scale synthesis of AuNRs using the El-Sayed protocol by (i) increasing the concentration of gold by a factor of 2; (ii) increasing the volume of aqueous solution by a factor of 600 (from 10 mL to 6 L), and (iii) increasing the yield in reduced gold to ca. 100 by slow addition of AA after the first growth step [65].

### 2.2. Silica Coating of Gold Nanorods (AuNR@SiO_2_)

Silica is widely used as a coating material for AuNRs. The benefits of silica coating are multiple, among them: An improved colloidal and thermal stability, an increase of the rods surface area while preserving the optical properties of the gold core, and the adjunction of a controllable porosity. Moreover, silica improves AuNRs biocompatibility, while its reactive surface silanols enable drug loading and surface conjugation by functional ligands or biomolecules for an efficient and selective probing of targeted molecules. In addition, SiO_2_ shell determines the refractive index of AuNRs surrounding environment, thus, thickness control is a crucial parameter affecting the LSPR response and sensitivity.

**Figure 5:**
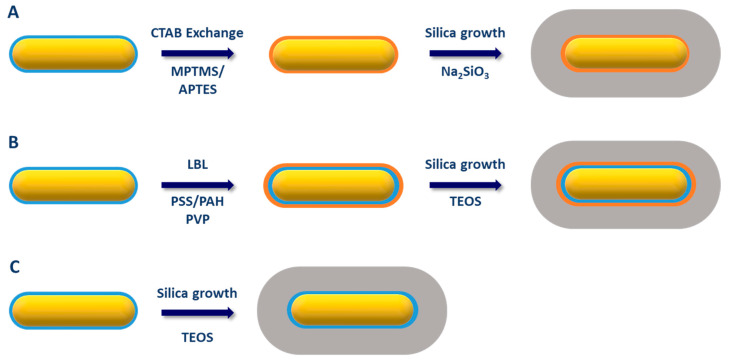
Strategies commonly used for AuNRs capping by silica. (**A**) CTAB exchange by a functional primer followed by silica growth, (**B**) coating through a primer on top of CTAB bilayer then silica growth, and (**C**) direct coating of silica on CTAB-stabilized AuNRs.

In the following subsection, we discuss the variety of synthetic methods that have been developed to encapsulate AuNRs with silica. Three possible strategies depicted in Figure 5 are utilized for AuNRs capping by silica: CTAB exchange by a functional primer, coating through a primer on top of CTAB bilayer, and direct coating. We also list other protocols such as microemulsion and biphasic growth at the end of this section.

#### 2.2.1. CTAB Exchange by a Functional Primer

Due to the strong interaction between CTAB and AuNRs surface and also to the stabilizing role that CTAB plays on colloidal AuNRs suspensions, its replacement by other molecules through ligand exchange is a complex reaction. The first reports on the coating of AuNRs with silica relied on priming the metallic surface with silane coupling agents or polymers to increase the affinity between gold surface and silica. These protocols are derived from the one developed by Liz-Marzàn et al. for citrate-stabilized gold nanospheres, in which the gold surface is modified, in water, by exchanging citrate ligands by (3-aminopropyl)trimethoxysilane (APTMS), a silane coupling agent.

**Figure 6:**
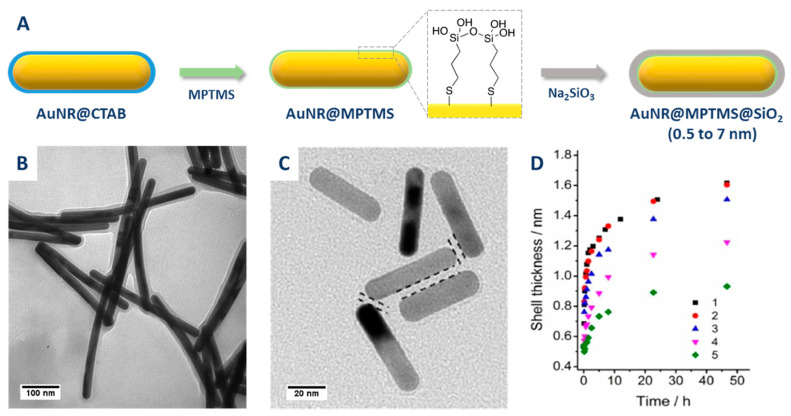
(**A**) CTAB replacement by (3-mercaptopropyl)trimethoxysilane (MPTMS) for the growth of thin layers of silica. (**B**) and (**C**) TEM images obtained for different aspect ratio (AR) from ref [67] (**B**) and ref [69] (**C**). (**D**) Kinetics of silica shell thickness for decreasing concentrations of Na_2_SiO_3_ from ref [69].

APTMS creates a silanol-rich surface on the AuNR, that favors the deposition of a silica layer by condensation of sodium silicate at pH = 10–11 [66]. In 2001, Murphy and co-workers reported the encapsulation of high AR AuNRs with a thin (5–10 nm) silica shell using (3-mercaptopropyl)trimethoxysilane (MPTMS) instead of APTMS (Figure 6A,B). This primer was chosen because of the stronger affinity of sulfur toward gold enabling the displacement of strongly adsorbed CTAB molecules [67]. Pérez Juste et al. applied a similar procedure to AuNRs with short AR (Figure 6C). They observed the formation of a thin but irregular silica shell (thickness 5–7 nm) and highlighted the role of CTAB micelles in the nucleation of free silica and as a consequence the importance of particle washing prior to silica encapsulation [68]. Later on, Li et al. reported on the formation of ultra-thin silica shell (UTSC) on MPTMS-capped AuNRs. The thickness of the silica shell was finely tuned in the 0.8–2.1 nm range by changing the amount of Na_2_SiO_3_ [69]. These authors also investigated the rate of silica-shell growth using LSPR. They observed an increase in the constant rate of formation of the silica shell upon increasing the Na_2_SiO_3_ concentration that was attributed to a rise of pH (from ca. 7 to ca. 9). A consequence of this high condensation rate of silica was the formation of core-free silica nanoparticles at high Na_2_SiO_3_ concentration. To avoid the formation of these undesired silica aggregates, a silica-shell precursor was added in a stepwise matter for the highest Na_2_SiO_3_ concentrations. Using this stepwise protocol, a thicker (up to 3.5 nm) silica shell was obtained. The texture of the silica shell grown using thiol-silane as primer, to the best of our knowledge, has never been characterized using N_2_-sorption. Nevertheless, it is usually considered as non-porous. Indeed, on the basis of Raman spectroscopy analysis of AuNR@SiO_2_ exposed to pyridine that strongly adsorbs on gold and not on silica, Li et al. have concluded to the absence of pinholes (exposing gold surface to reactants) in the shell of Au@SiO_2_ composites with a shell thickness above 4 nm [70].

Table 1 summarizes the reaction conditions and the silica shell aspects obtained when using a silane primer for silica growth. The source of silica is exclusively sodium silicate and the thicknesses are very low, ranging from 0.5 to 8 nm. Silane strategy is an effective way to grow, in aqueous solution, a thin silica layer shell on AuNRs surface. However, such protocol requires an extended time duration from one to six days for the silica shell to form and stabilize the particles (see Table 1), unlike the next primers we will be discussing.

**Figure 7:**
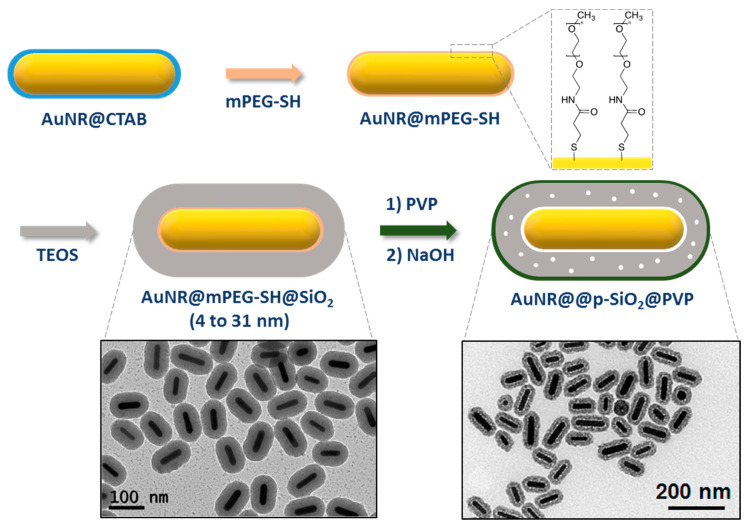
CTAB replacement by (O-[2-(3-mercaptopropionylamino)ethyl] O’-methylpolyethylene glycol (mPEG-SH) for silica growth and further induced porosity with the corresponding TEM images from ref [71,72].

Fernandez-Lopez et al. used a ligand exchanged-based protocol but with a thiol-modified poly(ethylene glycol) ((O-[2-(3-mercaptopropionylamino)ethyl] O’-methyl-poly(ethylene glycol, mPEG-SH) [71]. The action of this primer is similar to the one described above for the thiol-silane: The thiol function interacts strongly with the gold surface while the oxygen-rich poly(ethylene)glycol favors the nucleation of the silica shell. Ligand exchange is performed in water with an excess of mPEG-SH (150 molecules/nm^2^) and a minimum of CTAB (~1 mM). After this step, particles are washed to remove free CTAB micelles and excess of reactant and then transferred in a NH_3_/water/EtOH mixture for silica coating by the Stöber method. The resulting particles exhibit a non-porous shell of very well controlled thickness, which can be tuned between 4 and 31 nm by adjusting the amount of added tetraethyl orthosilicate (TEOS) (Figure 7). Surface composition of mPEG-SH functionalized AuNRs in classical conditions is expected to contain both mPEG-SH and CTAB molecules. In order to fully remove cytotoxic CTAB, Kinnear et al. proposed a two-step procedure that enhances PEGylation: The first step is performed by adding 10 mPEG-SH/nm^2^ to the AuNRs solution in water with ~1 mM CTAB and complete functionalization of the AuNR surface is secondly performed in ethanol/water solution (90% *v*/*v* EtOH in water) containing another 10 mPEG-SH/nm^2^. The use of EtOH is reported to increase the critical micellar concentration (CMC) of CTAB, hence destabilizing the bilayer and facilitating its desorption [73].

The use of PEG-SH/Stöber process requires shorter reaction times as reported in Table 1. In addition, while silica shell grown using PEGylation/Stöber process is essentially nonporous, Wang et al. recently reported the use of NaOH as etching agent to induce porosity in the silica shell. The silica outer layer is first wrapped with PVP to prevent complete dissolution of the shell, then by adjusting NaOH concentration and etching time, different structures were obtained (Figure 7). Surface area, pore volume, and average diameter were determined by N_2_ physisorption and estimated to be 183 m^2^.g^−1^, 0.32 cm^3^.g^−1^, and 3.2 nm, respectively [72].

#### 2.2.2. Coating through a Primer on Top of CTAB Bilayer

**Figure 8:**
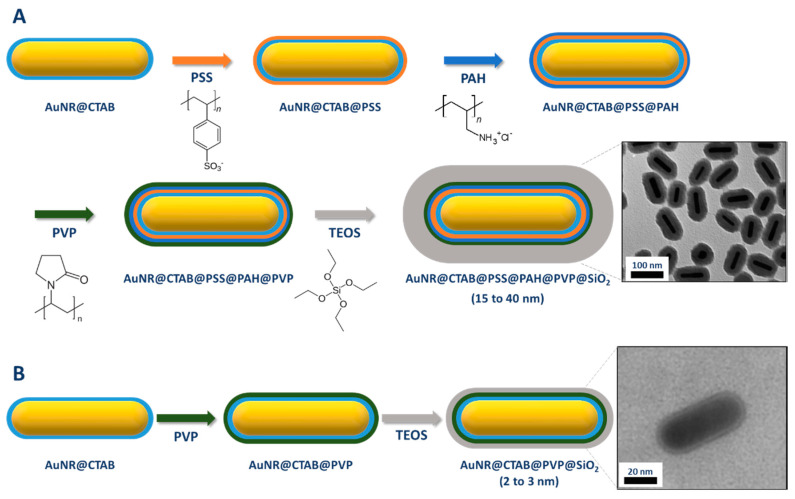
Silica coating on top of CTAB using an layer-by-layer (LBL) approach adapted from ref [75] (**A**) and ref [77] (**B**).

Graf et al. proposed in 2003 a very general protocol based on the intermediate adsorption of a layer of polyvinyl pyrrolidone (PVP) for the coating of colloidal nanoparticles with silica [74]. The role of this intermediate layer is to allow the transfer of water stable colloidal nanoparticles in the ethanol/H_2_O/NH_3_ solution used for classical Stöber process. However, according to Pastoriza-Santos et al., this protocol cannot be directly applied to CTAB-capped AuNRs. These authors also mentioned the difficulty to apply the protocol proposed by Murphy and coworkers to AuNRs with small AR (because of tip-to-tip aggregation of NR after ligand exchange of CTAB with the thiol silane). Therefore, they developed an alternative protocol whose purpose is to screen the CTAB molecules [75,76]. This screening is obtained by the layer-by-layer (LBL) adsorption of charged polyelectrolytes (Figure 8). Typically, AuNRs were successively wrapped in aqueous media with a negatively charged polystyrene sulfonate (PSS) and positively charged polyallylamine chloride (PAH). Finally, a last layer of PVP was added in order to reduce the surface charge (zeta potential below 20 mV). This reduction of the surface charge is required to preserve the colloidal stability of the AuNRs during the last step, which is the growth of a silica shell by base-catalyzed hydrolysis and condensation of TEOS in a 2-propanol/water mixture (Stöber process). Between each functionalization step, the particles are washed to remove excess reagents as well as free CTAB micelles leading to the formation of a non-porous shell. The control of the shell thickness from 15 to 40 nm (Table 2) was achieved by modulating the amount of TEOS added. Although efficient, this procedure may be laborious and time consuming because of the multistep process. Moreover, reaction conditions such as polyelectrolyte concentrations and molecular weight as well as the ionic strength of the solution should be carefully controlled because of potential risk of particle aggregation or heterogeneous silica shell.

More recently, Nallathamby et al. achieved PVP-mediated silica coating of CTAB-capped AuNRs, without the need of intermediate polyelectrolyte layers. Using this protocol, they obtained AuNR coated with a thin and homogeneous silica shell whose thickness was modulated by changing the PVP molecular weight [77].

#### 2.2.3. Direct Coating of AuNR with a Mesoporous Silica Shell

Direct coating of AuNR with silica was achieved by several groups as summarized in Table 3. Table 3 also reports the reaction conditions and the respective silica shell size and aspect.

Gorelikov et al. [78] were the first to report the direct coating of AuNR with silica. A detailed description of the synthesis protocol can be found in ref. [76]. The protocol is quite simple. Briefly, a solution of the CTAB-capped nanorods redispersed in water is basified to pH = 10–11 and a solution of TEOS in an alcohol (methanol, ethanol, etc.) is added to this solution under stirring. The suspension is then aged during several hours to days. As mentioned above, the initial attempts to directly coat the AuNR with silica failed and led to the formation of free silica nanoparticles, while leaving the AuNR uncoated. Gorelikov managed to solve this issue by simply adding a centrifugation step between the synthesis and coating steps. The purpose of centrifugation is to drastically decrease the concentration of CTAB in the AuNR solution before adding the silica precursor, TEOS. A total removal of CTAB molecules must, however, be avoided because they stabilize the AuNR suspension by providing an electrostatic repulsion thanks to their positively charged headgroups. According to the protocol of Gorelikov et al., the CTAB concentration should be about 1.5 mM (slightly above the CMC in water). The negative impact of extensive washing of the AuNR was emphasized by Cong et al. who observed that repeating twice the washing step leads to aggregation of AuNR together with core-free silica particles [79]. The residual CTAB in the solution, by interaction with the negatively charged silicate species formed by basic hydrolysis of TEOS, leads to the formation of a porous shell made of disordered pores (with an estimated pore size, based on TEM images of about 4 nm). These good textural properties have been later confirmed and refined by Liu et al. based on N_2_ physisorption measurements (pore diameter: 2.6 nm, surface area: about 510 m^2^.g^−1^) [80].

The detailed mechanism leading to the capping of the AuNR by this mesoporous silica shell was later explored by several authors and the composition of the synthesis solution was altered in order to control the thickness of the silica shell. Parameters such as CTAB, alcohol, AuNR and TEOS concentrations, pH, and aging times have been analyzed and most of them have a strong influence, not only on the thickness, but also on the shape (continuous coating vs. lollipop and dumbbell coatings) of the silica coating.

**Figure 9:**
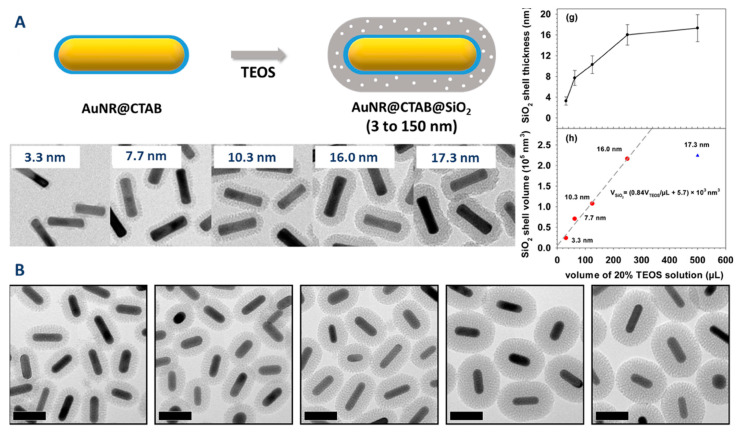
(**A**) Variation of silica coating thickness with amount of added TEOS: Left: TEM images (numerical values reported on each TEM are amount of TEOS and average coating thickness); right: Average thickness (top) and average volume (bottom) of silica coating vs. volume of added TEOS illustrating the complete conversion of TEOS to SiO_2_ shell up to 250 μL of 20% TEOS (red dots) and incomplete conversion above (blue triangle) (from [81]). (**B**) Variation of silica coating thickness with CTAB concentration: From left to right 1.2, 1.0, 0.9, 0.7, 0.4 (from [82]), the bar scale corresponds to 50 nm.

An expected increase in the silica shell thickness has been observed by several authors upon increasing the amount of added TEOS (Tian et al.: From 13 to 30 nm [86]; Wu et al.: From 3 to 17 nm [87] (Figure 9); Zhang et al.: From 3–38 nm [88]; Mohanta et al.: From 8–16 nm [89]). Wu et al. have also concluded that the thickness of the silica shell is, over a reasonable range of added TEOS, consistent with the full consumption of added TEOS (Figure 9). However, adding more TEOS either results in no modification in the thickness of the silica coating or, when a large excess of TEOS is added, to the formation of an irregular shell of silica, which only partially coats the gold nanorods.

Another less obvious parameter that is commonly used to modulate the thickness of the silica coating is the concentration of CTAB. Indeed, several authors observed that increasing the concentration of CTAB in solution results, for the same amount of added TEOS in thinner silica shell (Abadeer et al. [82]: From 26 to 11 nm (see Figure 9B); Yoon et al. [83]: From 30 to 13 nm).

Fine tuning of the thickness of the silica layer can also be, to a certain extent, achieved by modulating the duration of the synthesis. However, the addition of a terminating agent (PEG silane) before completion of the silica shell growth resulted in the production of a thin (as thin as 2 nm), smooth, and homogeneous shell [81], while quenching the reaction before completion leads to an inhomogeneous coating [90]. Other parameters have been less investigated such as the pH of the solution, but they also seem to play a role. For example, Huang et al. observed that decreasing the pH from 11 to 10.3 leads to dumbbell-shaped particles [90]. Another parameter whose influence has long been underrated is the amount of alcohol used to dilute TEOS. This parameter has been recently examined by Rowe et al. [84] They observed that, when the TEOS fraction in the TEOS/methanol solution was in the range 3–7 v%, a dumbbell-shaped silica coating was obtained (see below).

#### 2.2.4. Other Strategies

**Figure 10:**
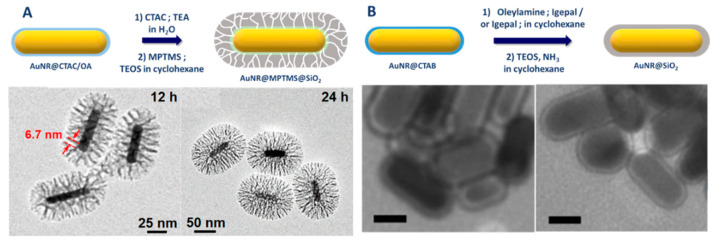
(**A**) AuNR@SiO_2_ with a highly porous silica shell grown in a biphasic solution [92]. (**B**) Silica coating of gold nanorods by reverse microemulsion from ref [77].

Other strategies relying either on a biphasic process or microemulsions were also utilized for silica capping of AuNRs. Xu et al. have proposed a modification of the thiol-silane/Stöber protocol, that allows growing a highly porous silica shell. In this biphasic process, inspired by the work of Shen et al. [91], MPTMS is added first to a AuNRs/TEA/CTAC (TEA: Triethylamine; CTAC: Cetyltrimethylammonium chloride) aqueous solution (25 wt% CTAC) and a solution of TEOS in cyclohexane is added on top of this aqueous solution [92] (Figure 10A).

A second strategy relying on microemulsion was also used for thin layer growth. Microemulsions are transparent and thermodynamically stable systems formed through the mixture of surfactant, water, and organic solvent (oil). The use of these homogeneous dispersions, especially the water-in-oil (W/O) or reverse microemulsion, is appealing to synthesize monodisperse nanoparticles or core-shell nanocomposites [93,94]. Reverse micelles dispersed in the continuous oil phase consist of water droplets stabilized by surfactant layer act as “nanoreactors” in which chemical reactions occur. In the case of silica coating, the cores are enclosed within the reverse micelles during the coating step thus preventing aggregation through steric stabilization, while the silica precursor (e.g., TEOS) is dissolved in the oil phase and hydrolyses at the water/oil interface. The major advantage of using reverse microemulsion is to restrict silica condensation to the limited water domain around the cores. This leads to the formation of dense and uniform silica coatings (shell thickness from 2 nm to tens of nm) on a variety of hydrophobic cores [95,96,97,98].

Reverse microemulsion is usually used to coat hydrophobic cores as they are readily dispersible and stable in the oil phase. As CTAB-capped gold nanorods are hydrophilic, they tend to aggregate in organic medium; so reverse microemulsion is not the favored method to coat gold nanorods with silica. Recently, Nallathamby et al. reported the silica coating of gold nanorods (AR of 3.6) by reverse microemulsions composed of IGEPAL/water/cyclohexane (IGEPAL is a trademark for polyoxyethylene (n) nonylphenyl ether). Their strategy and obtained TEM images are shown in Figure 10B. They reported that either hydrophilic CTAB-capped gold nanorods and hydrophobic oleylamine-capped gold nanorods (obtained after ligand exchange) can be, respectively, coated by a 3.8 nm and 6.1 nm thin silica shell [77].

#### 2.2.5. Commonly Encountered Difficulties and Their Remedies

- Core-free silica particles

Quite often TEM micrographs indicate the presence of undesired core-free silica particles. Yoon et al. proposed to proceed by successive injections of small amounts of TEOS (up to 16) in order to keep the concentration of silicate anions in solution below the nucleation threshold to avoid the formation of these undesired silica particles [83], while, for other authors, the formation of core-free silica nanoparticles may not be a problem because these particles are small (and light) and mostly remain in the supernatant during the centrifugation step [82]. Moreover, according to Wu et al., the formation of these core-free particles mostly occurs because the control of the thickness of the silica shell is obtained by increasing the CTAB concentration in the solution: Using conditions with low CTAB concentration and controlling the thickness of the silica shell by adjusting the TEOS amount allows converting all TEOS in shell coating of the AuNR [81].

- Dumbbell-shaped coatings

**Figure 11:**
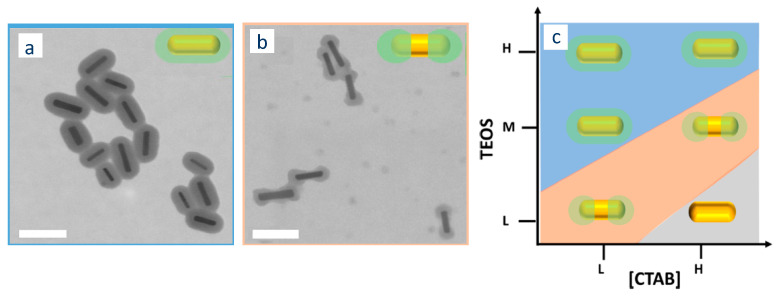
TEM images of AuNR@SiO_2_ obtained at constant TEOS concentration and with (**a**) 1 mM CTAB and (**b**) 9 mM CTAB. (**c**) Shape of the silica coating under various conditions according to Wang et al. [85].

Under certain experimental conditions, the silica shell grows exclusively/mostly on the two tips of the nanorods, leading to dumbbell/peanut-shaped coating (that can be either desired or undesired), see Figure 11. The origins of this shape are still not completely understood. According to Wang et al. dumbbell-shaped particles are formed because of the energy barrier that TEOS needs to cross to access the hydrophobic space located in the middle of the CTAB double layer that surrounds the AuNR. This energy barrier is stronger on the side of the AuNRs than on the tips (leading to a favorable nucleation of the silica coating around AuNR tips) and varies with the composition (concentration of TEOS, CTAB, and ethanol) of the reaction medium [85].

**Figure 12:**
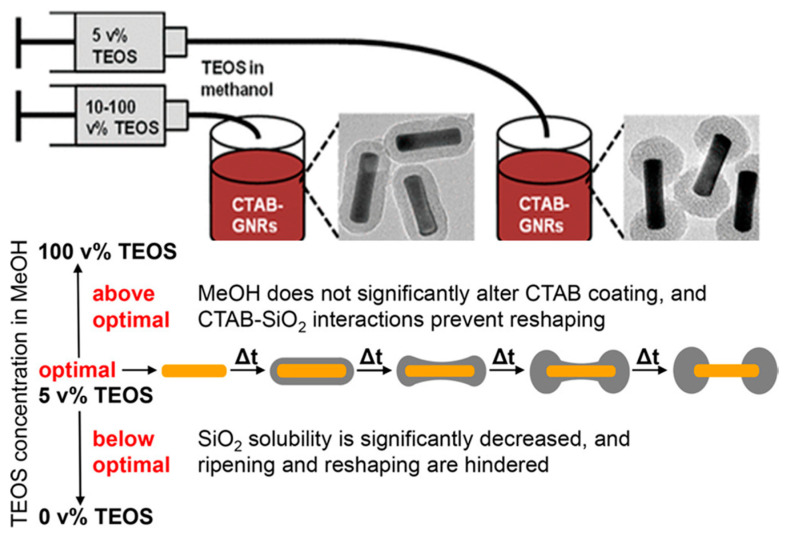
Top: TEM images showing continuous coating and dumbbell-shaped coating of Au NRs. Bottom: Cartoon showing the mechanism proposed by Rowe et al. to explain the influence of methanol on the formation of dumbbell shaped silica coating (from [84]).

Rowe et al. also investigated the influence of the amount of methanol on the shape of the silica coating (Figure 12). More precisely, they observed dumbbell-shell coating for TEOS-methanol compositions ranging from 3 to 7 vol% of TEOS (above and below these values, the silica shell forms a continuous and homogeneous coating). They also concluded, based on the shape of the coating after a short reaction time, that SiO_2_ is probably initially deposited all over the surface of the nanorod and that a reshaping of the coating from continuous to dumbbell occurs with time.

- Removal of CTAB

AuNR@SiO_2_ prepared using this type of protocol can still contain a fairly large amount of CTAB, as CTAB molecules are located not only at the AuNR surface, but also in the mesopores of the silica shell. The best-suited protocol to fully remove CTAB from the pores of the silica shell, while avoiding shape-transformation of AuNRs is, according to Feng et al., an extraction of CTAB using a NH_4_NO_3_/methanol solution [99].

#### 2.2.6. Concluding Remarks

This brief overview of the literature on the coating of AuNR with porous silica reveals that it is possible, with this method, to prepare silica-coated AuNR free of core-free silica and with a fine control of the thickness of the silica coating. It also shows that a complete mastering of the coating does require some expertise: (i) Tuning the thickness of the silica coating through an adjustment of the concentration of CTAB may lead to the formation of undesired core-free silica particles, and, although most of them can be eliminated by an appropriate centrifugation step, it is not unusual that a small fraction remains in the final sample; (ii) prediction of the thickness of the silica coating based on the amount of added TEOS is made difficult by the fact that the concentration of gold nanorods is not always precisely known. Moreover, parameters leading to a thin coating of AuNR with silica are close to those leading to a dumbbell-shaped coating.

## 3. Methods of Characterization

Several common techniques are used to characterize the structure and shape of gold nanorods. In the following, the main ones will be described and, for each of them, the possibility of characterizing an outer silica shell will be discussed.

### 3.1. UV-Visible Spectroscopy

**Figure 13:**
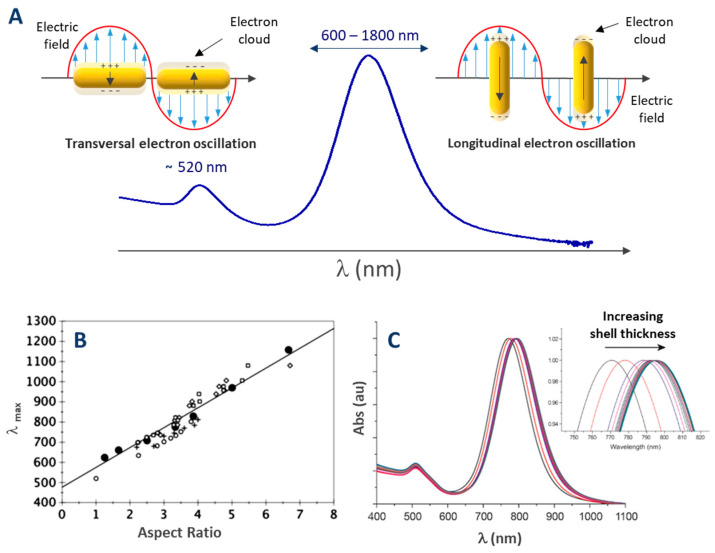
(**A**) Typical extinction spectrum of AuNR and the schematic representation of the electronic oscillations adapted from ref [103]. (**B**) Position of l-LSPR band maximum λ_max_ as a function of AuNRs AR, Simulation results using the DDA (Discrete Dipole Approximation) method (black circles) [14] and experimental data from the works of Al-Sayed et al. (open circles) [104], Pérez-Juste et al. (diamonds and squares) [105] and Brioude et al. (crosses) [14]. (**C**) l-LSPR band shift with increasing silica shell thickness adapted from ref [83].

**Figure 14:**
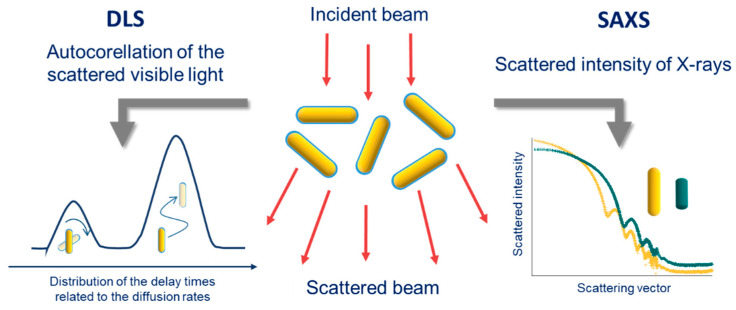
Scattering techniques commonly used to characterize AuNRs.

Gold nanoparticles are well known and used for their absorption in the visible range that is characteristic of LSPR. The associated resonance frequencies are related to the characteristic dimensions of the particle. In the case of gold nanorods, their two dimensions lead to two absorption bands in their UV-visible spectrum [100]: One due to the transverse localized surface plasmon resonance (t-LSPR) and one to the longitudinal localized surface plasmon resonance (l-LSPR) (see Figure 13A). If both are characteristic of the gold nanorods, l-LSPR is the one that is used and studied for nanorods. For example, because the l-LSPR band is subjected to shifts (usually a red-shift followed by a blue shift, see section Figure 13A) when the nanorods grow, it allows a simple monitoring of the growth of the rods [101]. However, this technique cannot be used as an absolute characterization of gold nanorods, as the position of the l-LSPR band is in fact correlated to the AR of the rods and not directly to their length (Figure 13B, [14]). As emphasized by Scarabelli et al. [42], other qualitative and quantitative information can be extracted from the UV-Vis spectrum:

1. The absorbance at 400 nm can be used for the quantitation of reduced gold (see details in the section of this chapter dedicated to quantitation);
2. The intensity ratio between l- and t-LSPR bands is a good qualitative indication of the polydispersity (a high ratio indicates a low polydispersity in the dimensions of the nanorods);
3. A shoulder close to the t-LSPR peak indicates the presence of AuNPs of other shapes (e.g., spherical AuNPs);
4. The width and the symmetry of the l-LSPR band are related to the polydispersity of the sample (a larger width and/or an asymmetric shape indicates a higher polydispersity).

These pieces of information are, however, only qualitative because the broadening of the l-LSPR band has also been shown to be related to the dimensions of the rods [102].

In addition, the position of the l-LSPR peak is extremely sensitive to the adsorption of molecules [106]. This feature is at the origin of LSPR biosensing as it will be discussed in Section 5. As a consequence, the position of the l-LSPR band also allows silica shell growth monitoring. Indeed, a red shift in its position has been observed in several studies and correlated to the thickness of silica shell up to a certain value [83]. Figure 13C illustrates the l-LSPR shift upon successive additions of TEOS resulting in silica shell thicknesses ranging from 8 to 20 nm.

### 3.2. Electronic Microscopy

As illustrated throughout this manuscript, electron microscopy is one of the most useful techniques to characterize gold nanorods. Indeed, it provides direct images of the nanorods. With transmission electron microscopy (TEM), the difference between the contrast of the gold core and the silica shell allows to characterize them separately. The quantitative analysis of micrographs displaying enough particles provides the distribution of the nanorod dimensions. Yet, unbiased and reproducible analysis of the micrographs is not an easy task as Grulke et al. showed in their work where they propose a standardized protocol to analyze such images [107].

The main limitations of electron microscopy characterizations are:

1. The handling of dried samples to analyze them in vacuum in the microscope chamber can also introduce multiple artefacts;
2. The selection of a necessarily limited number of micrographs of the samples can introduce a biased overview of the sample or, at least, a measurement uncertainty. General statistics like Sturges’ rule [108] or specific studies dedicated to nanoparticle distributions [109] can help to evaluate the accuracy of this limited sampling. These problems are amplified by the difficulty of the manual or automatic analysis of the dimensions of such objects on micrographs;
3. It is usually advised to analyze 200–300 nanorods to obtain a reasonable evaluation of the average dimensions and polydispersity in size of the nanorods, which can be time consuming if performed manually with a software such as ImageJ and automatic analysis is often not efficient (especially when particles of different shapes are present). New developments in this field are oriented toward the automatic analysis of TEM images by the development of appropriate algorithms [110].

To overcome these obstacles, another type of measurement can be performed, using some properties of the waves scattered by nanorods.

### 3.3. Scattering Techniques

Two main scattering techniques are commonly used to characterize gold nanorods: Dynamic light scattering (DLS) and small angle X-ray scattering (SAXS) (Figure 14). Both techniques operate in liquid and allow to analyze a large number of particles simultaneously, which provides, in general, a faithful description of the samples.

#### 3.3.1. DLS

DLS measurement is relatively easy to perform and this technique is commonly found in laboratories that deal with soft matter. Indeed, nanorod dispersions can most of the time be directly analyzed, although sometimes after dilution, and a quick and automatic measurement gives a result within a few minutes, provided that the laser used does not display a similar wavelength as one of the LSPRs of the nanorods. However, interpreting the results of this technique is not straightforward for anisotropic objects.

Indeed, DLS is based on the analysis of the dynamics of the loss of scattered light autocorrelation due to the Brownian motion of the studied particles. In other words, it consists of measuring the characteristic time taken by the particles to rearrange in a completely different random pattern that yields a different scattered intensity. In the case of isotropic particles, this time can be linked to their size through their diffusion constant and the Stoke–Einstein equation. When it comes to anisotropic particles, two characteristic times arise that correspond to two motions: Rotational and translational (Figure 12) [111]. These two times give rise to the observation of two signals in the particle size distribution, one of low intensity, at relatively low dimensions (a few nm) and the other at larger dimensions. The low intensity peak should not be mistakenly interpreted as indicating the presence of small particles, as it is actually due to the rotational diffusion of nanorods, while the larger peak at higher dimension is associated with translational diffusion [112].

Experimentally, using a system of polarizer/analyzer, it is possible through depolarized DLS (DDLS) to distinguish both contributions [113,114]. Several models and theories have been proposed to reproduce the experimental observations, but they are still debated [114]. Therefore, if these measurements are indubitably characteristic of the nanorod structure, they can mainly be used as a signature of the colloidal stability of the nanorod dispersions but not to directly infer their dimensions. DLS can also be used to highlight the stages of NR modification as well as the formation of aggregates: In particular, an increase in the peak corresponding to translational diffusion, if it is associated with the disappearance of the rotational scattering peak, indicates the formation of isotropic aggregates, whereas if it is associated with an increase in the intensity of the rotational scattering peak, it indicates a tip-to-tip aggregation of NRs [112].

#### 3.3.2. SAXS

Another relevant scattering technique to study nano-objects is small angle X-ray scattering (Figure 14). It is less commonly used as the corresponding setups are rarely present in laboratories and the synchrotron beamlines displaying this technique can only be accessed upon acceptance of a proposal. The use of SAXS seems perfectly relevant to characterize nanorods though, as the high density of gold gives a strong contrast with the dispersing phase. Hence, especially with synchrotron light sources, measurements can be performed quickly enough, to follow the growth of nanorods in real time [101,115]. The main limitation of this technique to characterize the length of rods is the smallest scattering vector q_min_ (corresponding to the smallest probed angle) that can be measured with the equipment that is used. Indeed, q_min_ is related to the biggest size that can be characterized: l_max_ ≈ 2π/q_min_. Hence, two rods displaying the same radius but different lengths, both superior to l_max,_ would not be distinguished.

Once obtained, the scattering curves need to be fitted with suitable model form-factors to determine the dimensions of the rods [116]. To precisely model nanorods, some authors had to use a combination of form-factors [117]. To take into account interactions between nanorods, a structure-factor can also be combined to the form-factor [118]. Unfortunately, very thin silica coating cannot be efficiently characterized through this technique, as its scattering contribution is not significant enough, but thicker layers should be quantifiable if modelled with the appropriate form factor, as well as more complicated structures (e.g., core/satellite superstructures in [119]).

### 3.4. Other Characterizations

#### 3.4.1. Zeta Potential Measurement

**Figure 15:**
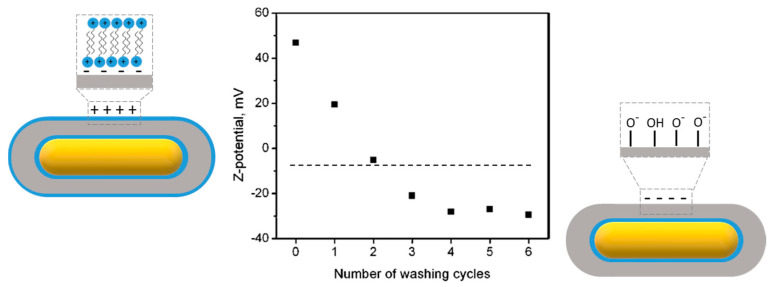
Monitoring CTAB removal by zeta potential measurements, adapted from [78].

Zeta potential measurements (usually based on electrophoretic mobility measurements) can be a precious help to monitor the changes that take place at the surface of the nanorods. Indeed, this technique gives indirect information about the surface charge that is strongly correlated with the type of material at the surface (gold, silica) and the molecules adsorbed at the interface (surfactants, polyelectrolytes, functionalization agents). This technique can be helpful to monitor the formation of a silica shell onto gold nanorods and also to follow their functionalization. For example, Gorelikov and Matsuura used it to monitor the effect of washing cycles on the gradual removal of CTAB surfactant from the surface of silica-coated gold nanorods (Figure 15) [78].

#### 3.4.2. Quantitation of Reduced Gold in AuNRs

Spherical gold nanoparticles are obtained under strong reduction conditions, leading to a complete reduction of Au^3+^ to Au^0^ and, as a consequence, a 100% reduction yield can be postulated. However, this is not the case for the preparation of AuNRs. Indeed, their synthesis requires the use of weak reducing agents, thus the hypothesis of a 100% reduction of the gold salts no longer applies. Therefore, the determination of the reduction yield (i.e., efficiency of gold incorporation into the AuNR) is crucial information for the optimization of the synthesis protocol, the investigation of AuNRs formation mechanism, the surface modification procedure, and some applications. As mentioned above, the reduction yields are still scarcely reported in the literature. The main techniques utilized for these characterizations are inductively coupled plasma coupled to optical emission spectroscopy or mass spectrometry (ICP-OES/MS), UV-Vis spectroscopy, and X-ray absorption near edge spectroscopy (XANES) measurements. ICP-OES/MS are highly sensitive but destructive elemental analysis techniques (AuNRs samples are digested in strong acids before analysis) that have been used to determine Au^0^ concentration in AuNR dispersions [46,120,121,122]. The extinction coefficient of l-LSPR band could also, in principle, be used for the quantitation of AuNRs concentration, provided that the value of the extinction coefficient at each wavelength can be calculated. Orendorff et al. [46] found that extinction coefficients of AuNRs depend on their AR (calculated from TEM images) and, as a consequence, on the energy of their l-LSPR band. Park et al. [122] later came to a different conclusion, which is the fact that the extinction coefficient of AuNRs depends mostly on the effective radius of the AuNR (R_eff_ = (3V/4π)^1/3^) and only marginally on its AR. However, the extinction coefficient is not only linked to the dimensions of the AuNRs, as dispersions containing particles with similar average dimensions but different polydispersities display significantly different absorptions [42]. To determine the concentration of reduced gold based on UV-Vis spectroscopy, several authors have proposed to monitor the absorbance at 400 nm, assuming that interband transitions of Au^0^ at this wavelength are the only contribution to the absorption and are independent of the particle size, shape, and surface chemistry [42,123,124,125]. Edgar et al. found a reliable correlation (within 20% discrepancy) in Au^0^ concentration using ICP-MS or optical measurement by analyzing the data published by Orendorff et al. [126]. Scarabelli et al. [42] found a linear correlation between absorbance at 400 nm and the concentration of reduced gold in solution and established that a value of 1.2 for the absorbance at 400 nm corresponds to a Au^0^ concentration of 0.5 mM. Hence, UV-vis spectroscopy can be used for quantitation of reduced gold with a relatively good precision with the added advantage that it can be used in situ. Finally, XANES studies were also conducted to monitor the Au^3+^, Au^+^, and Au^0^ concentrations and their incorporation during AuNR growth as this technique allows to discriminate the different redox states of gold [101,115]. However, the limited accessibility to a synchrotron X-ray source necessary for this kind of measurement remains a major drawback for routine analysis.

#### 3.4.3. Silica-Shell Porosity Assessment

The porosity of a 20 nm-thick mesoporous silica shell coated on AuNR was directly measured by N_2_ physisorption analysis and was found similar to that of mesoporous silica nanoparticles templated with the same CTAB surfactant. Porosity was also indirectly probed by SERS with aromatic thiols of increasing bulkiness. Owing to the strong dependence of the SERS signals intensity on the distance to the gold surface, only the molecules smaller than the pores and able to penetrate within the silica core can chemisorb to the gold surface and will then provide a Raman signal. The absence of a Raman signal for the AuNR@SiO_2_ exposed to the largest thiol of the series indicated that the molecule was unable to reach the bottom of the mesopores but rather adsorbed to their walls [127].

## 4. Functionalization Methods of Gold Nanorods

Prior to their use for biosensing, AuNRs require a functionalization step during which the biological element responsible for target recognition becomes attached to the particles. The literature survey shows that three general strategies are employed for this purpose, namely physisorption, chemisorption, or conjugation.

### 4.1. Physisorption

Physisorption is by far the easiest method to immobilize a bioreceptor on AuNR. Conversely to citrate-coated gold nanospheres, the organic capping of AuNR, namely the CTAB double layer, is strongly attached to the gold surface, thereby not easily displaced by competing molecules. The highly positive surface charge conferred by the CTAB layer enabled antibodies to be physisorbed at the surface of AuNR probably by electrostatic interaction [128,129]. Let us note that the long-term stability of the resulting nanoprobes has not been investigated.

Alternatively, the LbL technique, previously discussed in Figure 8, was applied to electrostatically physisorb antibodies to CTAB-capped AuNR in solution [130,131,132] or immobilized on a glass slide [133]. The principle of the LbL technique relies in the successive coating of negatively and positively charged polyelectrolytes to alternatively confer positive or negative surface charge to the nanorods. This procedure conveniently masks the cytotoxic CTAB layer and enables to cover the entire surface of the nanorods by the bioreceptor and not only the tips [130]. When coated by silica, the surface charge of nanoparticles becomes negative at physiological pH due to the low PZC of silica, allowing for an opposite electrostatic adsorption [134]. Antibodies have been physisorbed to negatively charged silica-coated AuNR [135]. Here, again, no study on the long-term stability of the resulting antibody-AuNR bioconjugates was performed.

### 4.2. Chemisorption

**Figure 16:**
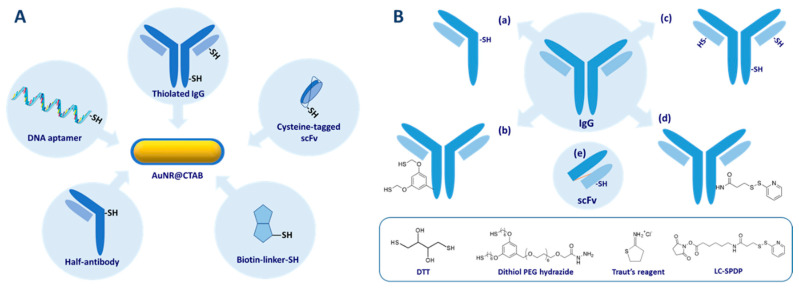
(**A**) Chemisorption of various thiol-containing biomolecules to CTAB-capped AuNR. (**B**) Introduction of thiol groups on IgG-type antibodies, (**a**–**d**), starting from an IgG using: (**a**) DTT (dithiotreitol); (**b**) NaIO_4_ then dithiol PEG hydrazide; (**c**) Traut’s reagent; (**d**) N-Succinimidyl-6-(3’-(2-PyridylDithio)-Propionamido)-hexanoate (LC-SPDP); and (**e**) scFv.

Another relatively straightforward strategy to conjugate bioreceptors to AuNR is to take advantage of the strong affinity of sulfur for gold, particularly in the form of thiols and disulfides. This approach has been employed to conjugate various thiol-containing bioreceptors including various thiol-terminated DNA oligomers [136,137,138,139,140,141,142,143], a cysteine-containing peptide [144] and a thiolated lactose derivative [145] to CTAB-capped AuNR in solution or immobilized on glass slides (Figure 16A). The anionic surfactant SDS can be added during chemisorption of aptamer to prevent NP aggregation [142].

Since IgG-type antibodies do not naturally contain any free cysteine in their sequence, several pathways have been used to create thiol groups (Figure 16B) prior to chemisorption to AuNR.

Initially, the heterobifunctional cross-linker N-Succinimidyl-6-(3’-(2-PyridylDithio)-Propionamido)-hexanoate (LC-SPDP) enabled the introduction of reactive disulfide bonds on an IgG type antibody [121]. Later on, Traut’s reagent (2-iminothiolane), a popular protein thiolation reagent was employed to introduce sulfhydryl groups by reaction of some of the amino groups of antibodies [146,147,148]. Occasionally, CTAB molecules capping AuNR were temporarily replaced by PVP and SDS [147]. Other strategies have been implemented to introduce sulfhydryl groups on antibodies in a site-selective fashion, away from the antigen binding site. For instance, selective reduction of disulfide bridges located at the hinge region of IgG using a mild reducing agent like DTT (dithiotreitol) afforded half-antibodies with 2 or 3 sulfhydryl groups [146,149]. Alternatively, the oligosaccharide residues located on the Fc domain of most IgG were mildly oxidized with NaIO_4_ to generate aldehydes followed by reaction with a dithiol PEG hydrazide heterobifunctional cross-linker [146,150]. Finally, a single chain Fv (scFv) fragment engineered to include a single cysteine tag was genetically produced and readily chemisorbed onto AuNR immobilized on glass slides [151]. Generally, the conjugation step is followed by a blocking step, typically with mPEG-SH, 11-mercaptoundecanol, or 6-mercaptohexanol, to prevent further nonspecific binding.

Owing to the known strong binding of CTAB to the AuNR, the question of complete or incomplete displacement of CTAB molecules by competing thiolated bioreceptors in solution arises. Regarding the chemisorption of thiolated lactose, it was clearly shown to be incomplete since the zeta potential of the nanorods remained positive after reaction [145]. The same conclusion was drawn in the case of an iminothiolane-treated IgG [148] and a thiol-terminated DNA aptamer [152].

To make sure all physisorbed CTAB molecules are properly displaced during ligand exchange and/or to prevent particle aggregation during the functionalization process, or even to make sure that the whole nanorod surface is covered with bioreceptor (and not only the tips, see below), more sophisticated procedures were put in place to chemisorb thiol-terminated DNA oligomers onto CTAB-capped AuNR.

**Figure 17:**
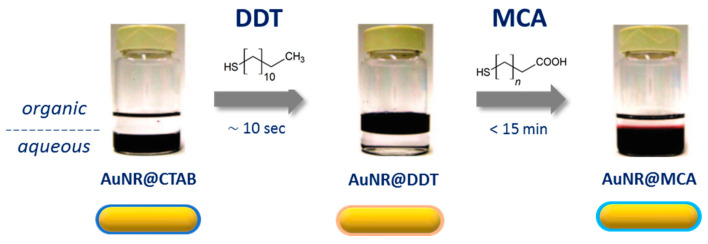
Round-trip” phase transfer method to prepare a DNA-AuNR bioconjugate [153].

A “round-trip” phase transfer method was introduced to prepare a DNA-AuNR bioconjugate (Figure 17) [153]. It relies on the exchange of CTAB molecules covering AuNR with mercaptoalkylcarboxylic acids followed by a second exchange with thiol-terminated DNA oligomer. The addition of dodecanethiol (DDT) to a suspension of CTAB-capped AuNR followed by the addition of acetone resulted in extraction of the NP in the organic phase while the CTAB molecules remained in the aqueous phase. After removal of excess DTT by addition of toluene and methanol, the NP were taken up in toluene and the solution was heated to 70 or 95 °C in the presence of mercaptoalkylcarboxylic acid until aggregation occurred. After washing and deprotonation with isopropanol, the NP were taken back into an aqueous phase where they were fully soluble. The mercaptohexanoic acid-coated AuNR were used to prepare a DNA-AuNR conjugate by exchange of the MCA ligand with 5′-thiolated DNA oligomer. Progressive addition of salt was necessary to prevent particle aggregation during DNA adsorption for charge screening. The final bioconjugate displayed very good stability over time and a fluorescence assay gave an average of 28 DNA strands per NP.

Gates and coll. reported an alternative two-step procedure where the initial CTAB layer is temporarily replaced by loosely bound PVP and SDS, which are in turn replaced by thiolated-ssDNA applying salt screening as above. The number of DNA strands per NP depended on the initial DNA/AuNR ratio and reached saturation at a ratio of ≥25,600:1 with a maximum loading of 870 ± 60 DNA strands per NP [154].

Another strategy involved the chemisorption of mPEG-SH in the presence of the nonionic detergent Tween 20 followed by addition of thiolated DNA and citrate to screen the charge repulsion between AuNR and DNA. Tween 20 helped to displace CTAB molecules and further stabilized the nanoparticles against aggregation. It was found that the DNA/AuNR ratio was inversely related to the initial amounts of mPEG-SH and Tween 20 used at the intermediate step. An average of 200 DNA strands per particle was determined by fluorescence measurement [155].

### 4.3. Conjugation

**Figure 18:**
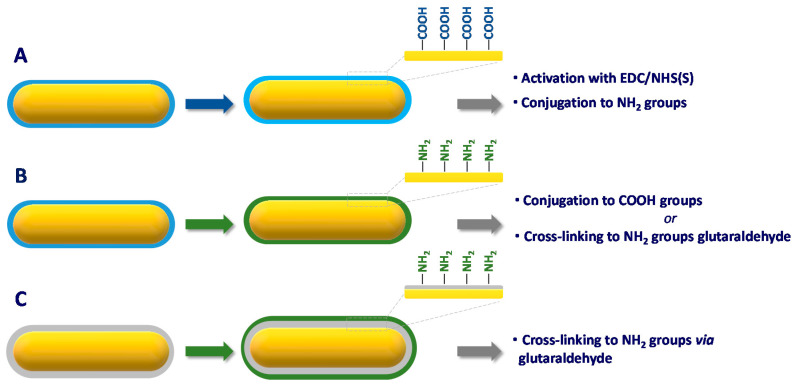
Covalent conjugation of bioreceptors to AuNR. (**A**) Grafting of carboxylic acids via ligand exchange; (**B**) grafting of primary amines via ligand exchange; (**C**) grafting of primary amines to silica-coated AuNR with (3-aminopropyl)trimethoxysilane (APTMS) or polyethylenimine (PEI).

Unlike simple chemisorption, and although it includes chemical bond formation, we refer to bioconjugation when the nanoparticles surface is modified and somehow tethered to attach the biomolecule. Two-step functionalization methods were also applied to attach various bioreceptors to AuNR and AuNR@SiO_2_ as illustrated in Figure 18.

**Figure 19:**
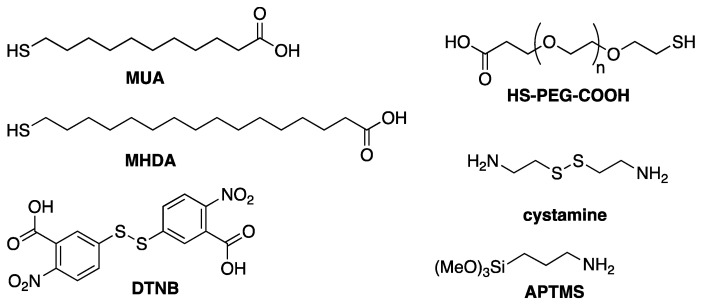
Bifunctional reagents for conjugation of bioreceptors to CTAB-capped AuNR and SiO_2_@AuNR.

The most popular method involves intermediate grafting of carboxyl functions at the surface of the nanoparticles, followed by coupling with reactive amine groups carried by the bioreceptor in the presence of a mixture of N-(3-Dimethylaminopropyl)-N′-ethylcarbodiimide (EDC) and N-Hydroxysuccinimide (NHS) (Figure 18A). Figure 19 depicts the structure of compounds used to introduce carboxyl groups on AuNR. A characteristic that have in common is to include a terminal thiol function to create strong Au-S bonds and a carboxylic acid function at the other end, typically MUA [20,156,157,158,159], MHDA [160,161], or mercaptoPEG acid derivatives [162,163,164,165,166,167]. This general strategy has been applied to decorate AuNR with bioreceptors both in solution or once deposited on solid substrates (essentially glass slides) using the mercaptocarboxylic acids alone or in mixture with other thiols like mPEG-SH or 11-mercaptoundecanol in various proportions. Occasionally, grafting of MUA was achieved by the “round-trip” phase transfer ligand exchange method [132,168,169] described in part 4.2. The disulfide derivative DTNB was also used to attach a peptide aptamer to AuNR via EDC/NHS coupling while providing a convenient Raman reporter for SERS biosensing [170].

Another route involved the reaction of cystamine (Figure 18B) with CTAB-capped AuNR to introduce reactive amine groups at the surface of gold. This strategy was used to graft biotin [171] and folic acid [172] via EDC/NHS activation or antibodies via glutaraldehyde cross-linking [173,174]. A similar strategy was employed to attach antibodies to silica-coated AuNR using APTMS to graft amino groups to the surface instead of cystamine (Figure 18C) [175].

### 4.4. Selective Grafting at the Ends or the Sides of AuNR

Selective grafting strategies take advantage of the shape and anisotropic features of gold nanorods. On the one hand, they allow to generate high regular superstructures of nanoparticles thanks to ligand-bioreceptor recognition (see below [176]). Alternatively, selective grafting of the ends of nanorods is motivated by the heterogeneous distribution of the electric field enhancement at the gold nanorods, with enhancement being more pronounced at the nanoparticle tips as compared to the side thus allowing highly sensitive optical detection down to the single molecule level [177].

It had been noticed that the molecules of CTAB located at the ends of gold nanorods were relatively labile and therefore more easily exchangeable by thiols or disulfides [178]. This feature made it possible to selectively attach bioreceptors to the tips of AuNR. The site-selective chemisorption of thiolated biotin molecules was achieved at the tips of AuNR in solution [179]. Similarly, biotin molecules were also chemisorbed at the tips of AuNR immobilized on glass slides silanized by MPTMS. This was performed by exposure of UV/ozone cleaned, AuNR-functionalized slides to a solution of CTAB to form a dense bilayer at the surface of the nanoparticles, followed by treatment with a solution containing both thiolated biotin and CTAB [177,180]. The latter was necessary to ensure site-selective grafting at the tips [181]. Following this procedure, it was estimated that the amount of biotin molecules at the tips of the AuNR was seven times larger than on the side [180]. Along the same line, 5′- and 3′-thiolated DNA oligonucleotides were site-selectively chemisorbed to the tips of AuNR by displacement of CTAB [182,183].

Yu and Irudayaraj used the same property to selectively attach IgG F_ab_ to the ends of AuNR through preliminary chemisorption of MUA followed by coupling via EDC/NHS. By choosing an initial F_ab_-to-AuNR ratio of 2, an average of ca. 1 F_ab_ per AuNR was grafted regardless of the AR [184].

**Figure 20:**
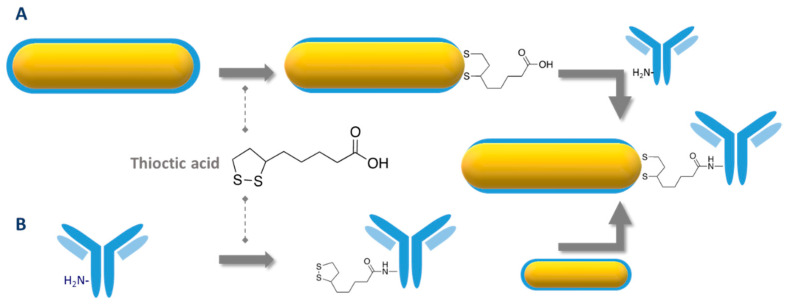
Tip-selective covalent conjugation bioreceptor to CTAB-capped AuNR. (**A**) Grafting of thioctic acid to AuNR followed by conjugation via N-(3-Dimethylaminopropyl)-N′-ethylcarbodiimide (EDC)/N-Hydroxysuccinimide (NHS); (**B**) coupling of thioctic acid to antibody via EDC/NHS followed by grafting to AuNR.

Kotov and coll. reported two original conjugation strategies to graft proteins at the tips or on the side of the gold nanorods [176]. Grafting at the tips was ensured by ligand exchange followed by covalent attachment of target proteins. In practice, carboxyl groups were predominantly introduced at the tips of AuNR by reaction of thioctic acid followed by MC-LR-OVA antigen or anti-MC-LR Ab conjugation by EDC/NHS coupling (Figure 20A). This procedure afforded a nanoprobe with an Ab/AuNR ratio of ca. 10. Such a site-selective functionalization had previously been reported by Chang et al. to selectively graft anti-mouse IgG antibody to the tips of CTAB-capped AuNR [185]. The authors also pointed out that preferential binding of thioctic acid was due to its cyclic thus rigid structure. A similar procedure was employed by Xu and coll. to site-selectively anchor MC-LR-OVA antigen or anti-MC-LR Ab to the tips of AuNR except that thioctic acid was conjugated to both proteins prior to chemisorption (Figure 20B) [186].

**Figure 21:**
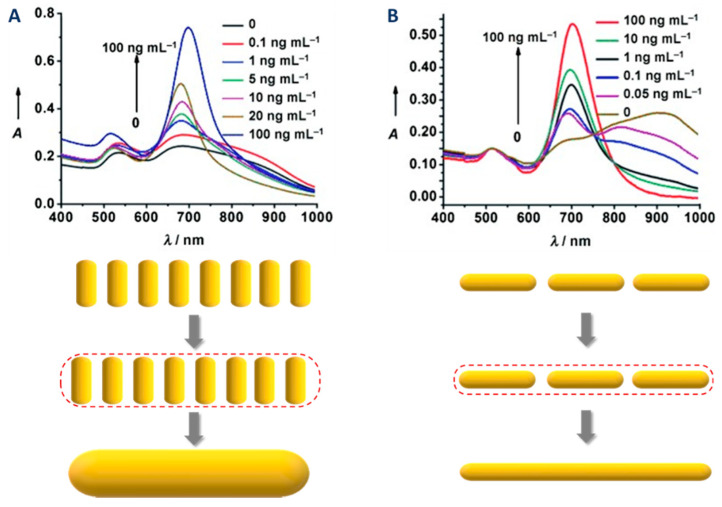
Evolution of surface plasmon resonance spectra of the nanorods upon increasing concentrations of microcystin-LR (indicated in the graph) and graphical representation of a plasmon system and corresponding nanowire approximation for side-to-side (**A**) and end-to-end (**B**) assemblies, from ref. [176].

On the other hand, selective grafting on the side of the rods was achieved by electrostatic binding of proteins, taking advantage of the larger area of contact affording stronger electrostatic interactions [176]. In this case, the Ab/AuNR ratio was estimated to be 31. When the two nanoprobes resulting from electrostatic binding of MC-LR-OVA antigen or anti-MC-LR Ab were mixed together, the rods assembled to predominantly form ladders (side-by-side assemblies; figure). Conversely, when the two nanoprobes resulting from covalent binding of MC-LR-OVA antigen or anti-MC-LR Ab were mixed together, the rods assembled to form strings (end-to-end assemblies; Figure 21). Interestingly both these assemblies were stable in the long term. The same strategy was used by Wang and coworkers to prepare two nanoprobes whose side was coated by gentamicin-OVA antigen and anti-gentamicin antibody [187].

Finally, a recombinant protein resulting from the fusion of staphylococcal protein A and gold binding polypeptide (GBP-SpA) was predominantly chemisorbed at the tips of CTAB-capped AuNR. Most importantly, activated charcoal was added to the reaction mixture in order to absorb released CTAB molecules and prevent particle aggregation. FT-IR spectroscopy analysis of the bioconjugate revealed the presence of remaining CTAB molecules on the nanoparticles [188].

## 5. Applications of AuNRs in LSPR Biosensing

**Figure 22:**
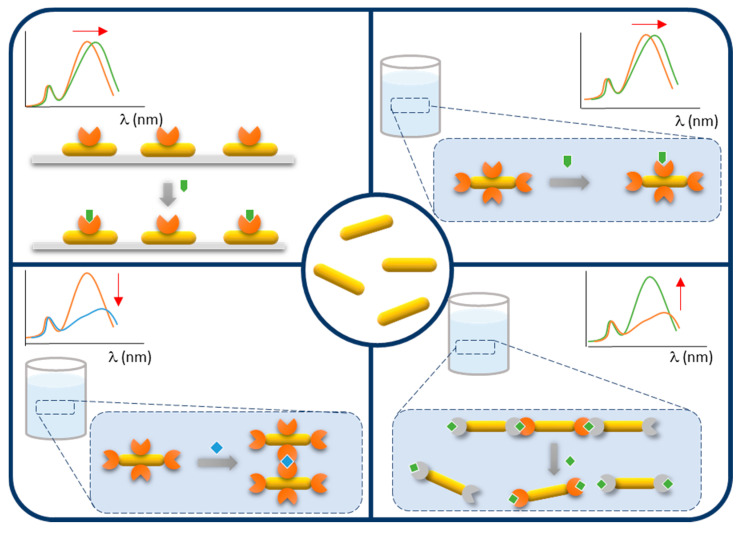
Summary of the operating configurations for Gold Nanorods nanoplasmonic biosensors.

An important asset of LSPR biosensors is the simplicity of the readout as the measurements can be done with a benchtop UV-visible spectrophotometer [189], an equipped smartphone [190], and sometimes even simply by naked-eye readout [12,191]. Gold nanorods plasmonic biosensors operate following four main mechanisms summarized in Figure 22.

In what follows, we recap the optical properties of AuNR in relation to LSPR biosensing, then survey the applications classified according to the biorecognition element. Most of the papers we review in this section deal with gold nanorods without silica coating. Indeed, up to now, only two examples of biosensors based on AuNR@SiO_2_ have been reported in the literature [135,175]. Both of them involve antibodies as bioreceptors and in one of the cases, it was demonstrated that the presence of a thin SiO_2_ shell not only provided a protective layer and increased the shelf life of the particles, but also improved the overall efficiency of the biosensor [135].

### 5.1. Optical Properties of AuNR in Relation to LSPR Biosensor Development

Gold nanorods possess unique optical properties owing to the localized surface plasmon resonance phenomenon [103]. They exhibit anisotropic plasmonic responses translated into longitudinal and transverse plasmon modes with extremely high extinction coefficient giving rise to bright colours from purple to brown owing to light absorption and scattering processes (Figure 13A). The position of the longitudinal plasmon band can easily be tuned from the visible to the near IR spectral range just by changing the AR (Figure 13B, [14]). Another extremely useful property of AuNR regarding LSPR biosensor development is certainly the sensitivity of the longitudinal plasmon wavelength to the refractive index of the surrounding medium.

The plasmon band shift ∆λ is governed by Equation (1),

(1) $$∆\lambda =m\left(n_{adsorbate}-\;n_{medium}\right)\times \left\{1-e^{\left(-\frac{2d}{l_{d}}\right)}\right\}$$

where n is the refractive index, *l_d_* is the decay length of the electric field, d is the thickness of the adsorbate layer, and m is the intrinsic RI sensitivity factor.

The parameter m can be experimentally determined by measuring the extinction spectrum of AuNR in aqueous solutions containing increasing concentrations of sucrose or glycerol. It also depends on the size and shape of the gold nanoparticles [192].

**Figure 23:**
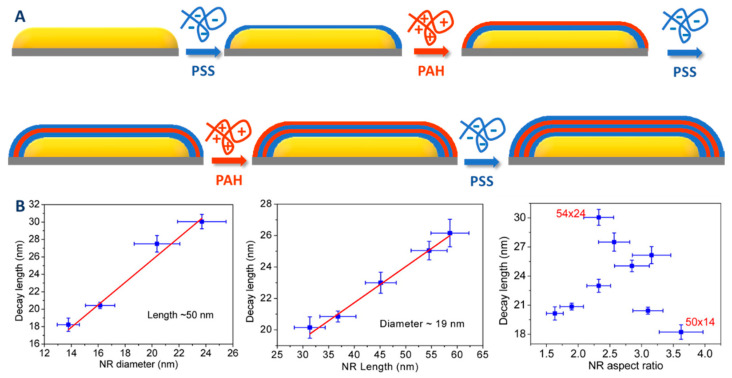
(**A**) Schematic representation of AuNR on a glass substrate covered with polyelectrolyte multilayers by the LBL approach. (**B**) Electromagnetic decay lengths of AuNR with different diameters, lengths and AR of AuNR, adapted from ref [194].

Another important property is the exponential decrease in electrical field enhancement making the plasmon band shift highly distance dependent (parameter *l_d_* in Equation (1)). This feature enables separation-free assays since only the events occurring in the neighborhood of the particles will contribute to the plasmon band shift. The electromagnetic decay length *l_d_*, defined as the distance from the nanorod surface at which the electric field enhancement is reduced by a factor of e [193], has been experimentally determined for AuNR of various sizes using a LbL approach [194]. The strategy adopted in this study consists in treating AuNR-coated glass slides with polycations and polyanions alternatively to create an LbL structure of increasing thickness while monitoring the position of the LSPR band (Figure 23). This enlightening study revealed that *l_d_* is linearly correlated to both diameter and length of the particles, but the *l_d_*/diameter slope is 5 times larger than the *l_d_*/length slope. For instance, an AuNR of 24 × 50 nm had a decay length *l_d_* of 30 nm. This value should be taken into account when designing an LSPR biosensor for a given target. On the whole, increasing AR results in red shifts in λ_max_, higher m, and longer *l_d_* [195].

Characterization of the sensing capabilities of metal nanostructures is best described by the figure of merit (*FoM*), which is calculated according to Equation (2) where FWHM is the full width at half maximum [192]:

(2) $$FoM=\frac{m}{FWHM}$$

The survey of the literature shows that LSPR biosensors including gold nanorods can be roughly classified into two categories, either solution phase- or solid phase-based configurations. They can also be classified according to the type of bioreceptor responsible for analyte recognition and capture. We will successively survey all these configurations.

### 5.2. Immunosensors

Immunosensors are the class of biosensors using an antibody as bioreceptor. Antibodies are essentially immunoglobulins G produced in various animal species to bind antigens with high affinity and specificity. IgGs are glycoproteins with a molecular weight of 150 kDa, a Y-shaped 3D structure, and an approximate size of 12 × 14 × 7 nm, comprising two antigen binding sites located at the two extremities of the F_ab_ domains. Their association to spherical nanoparticles has been widely studied [196]. AuNR-antibody bioconjugates operate either in homogeneous solution-phase or in solid-phase when deposited on planar substrates as discussed in what follows.

#### 5.2.1. Solution-Phase Based Immunosensors

**Figure 24:**
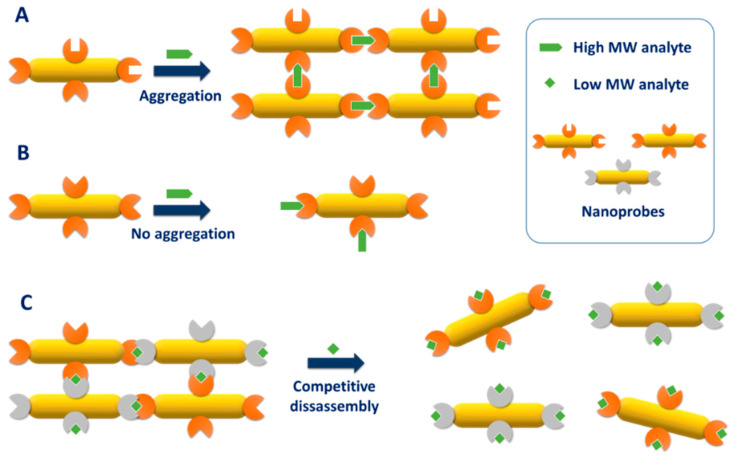
Immunosensor configurations. (**A**) Polyclonal antibody and high molecular weight analyte; (**B**) monoclonal antibody and high molecular weight analyte; (**C**) antibody and low molecular weight analyte.

In solution, the optical response of an AuNR nanoimmunoprobe to a given analyte depends on (1) the type of antibody and (2) the size of the analyte. For high molecular weight analytes such as proteins having more than one epitope, the use of a polyclonal antibody as bioreceptor results in nanoparticles aggregation, which translates into a decrease of the LSPR band intensity (Figure 24A), whereas a monoclonal antibody will yield a red shift of the LSPR band as a result of change of the local refractive index due to analyte binding (Figure 24B). LSPR biosensors for low molecular weight analytes having only one epitope are based on the assembly of antibody and antigen nanoprobes forming aggregates. Addition of analyte leads to a progressive disruption of the nanoparticle network, which translates into an increase of the LSPR band (Figure 24C).

Solution phase immunosensors have been applied to various analytes (Table 4) giving rise to biologically meaningful limits of detection. Signal enhancement was achieved by optimizing the distance between the analyte and the surface of the nanorods. For instance, Tang et al. showed that covalent binding of antibody to AuNR via conjugation to a self-assembled monolayer (SAM) of MUA significantly improved the sensitivity as compared to physically bound Ab via coating to PSS layer on CTAB [132]. This was rationalized by the shorter distance between the analyte and the rod surface that places it within the sensing volume. The same group also used magnetic nanoparticle (MNP) immunoprobe in conjunction to AuNR immunoprobe to both selectively extract cTnI and enhance the LSPR signal owing to the high RI of MNP [168]. Another way to enhance sensor response was to coat the nanorods with a thin layer of silica before grafting the antibody [135]. This resulted in an increase of RI sensitivity (parameter m in Equation (1)) and in turn to an extremely low LoD in the detection of the food pathogen *E. coli O157:H7*.

It has been shown that the LSPR associated electric field is anisotropically distributed, being largest at the ends of the rods with respect to the sides [13,180]. This feature was exploited to build up an immunosensor of the environmental toxin MC-LR [176]. For this toxin, having only one epitope, the competitive disassembly format was chosen and two pairs of nanoprobes were synthesized for which the binding partners (antigen and antibody) were grafted at the sides or on the ends of AuNR. In the absence of competitive analyte, the pairs of nanoprobes were assembled into ladder (sides) or chain (tips) patterns (see Figure 21), which were progressively disrupted upon addition of MC-LR. Remarkably, the sensor configuration based on end-to-end nanoparticle assembly displayed much better analytical performances.

As mentioned above, one of the most remarkable properties of AuNR is the ability to tune the position of the l-LSPR wavelength across a large range of the spectrum, i.e., from ca. 600 to 1000 nm by changing the AR of the rods. This property was exploited to set up multiplex assays, where each analyte is associated with a gold nanorod of a given AR so that overlap between the longitudinal band of each rod is minimal. In this way, a multiplex biosensor was reported for the simultaneous detection of two pathogenic bacteria [173] and two myocardial infarction biomarkers [169].

#### 5.2.2. Solid-Phase-Based Immunosensors

Solid phase AuNR-based immunosensors present several benefits with respect to solution-based ones. First they generally give higher RI sensitivity and FoM [159]; second, they enable real-time monitoring of binding events and therefore determination of kinetic and equilibrium constants [161]; third, they are easier to handle. Moreover, since the nanorods are firmly attached to their substrate, the formation of antibody–analyte pairs always result in a red shift of the LSPR band whose magnitude is dependent on analyte concentration until saturation occurs at full occupation of the binding sites. In the following, we will focus on bottom-up processes to prepare AuNR-based chips. However, it is important to note that several assays were performed with top-down generated LSPR-substrates [197], but as these platforms do not include colloidal particles in their engineering, they are out of the topic of this review and will not be included in the discussion below.

Construction of solid phase AuNR-based biosensors requires the immobilization of AuNR onto transparent substrates generally made of silica (glass slide). Adhesion of CTAB-capped AuNR to glass was performed in different ways either by (1) physisorption via electrostatic interaction to negatively charged piranha- (+ O_2_ plasma) or NaOH-treated substrates or (2) covalent bonding by silanization with APTMS or MPTMS to introduce primary amines or thiols, respectively, resulting in the formation of Au-N or Au-S bonds. Alternatively, thiol groups have been introduced by sequential reaction of APTMS, succinic anhydride, EDC/NHS, and cysteamine [151]. Immobilization of AuNR@SiO_2_ on quartz slides was achieved by coating of the substrates with PVP [175]. Grafting of the antibody to the AuNR is then performed by one of the methods described in part 4. Occasionally, the reverse process by which the antibody is first grafted onto the AuNR in solution then the conjugate is immobilized onto glass slides treated with APTES and glutaraldehyde was also performed [164].

In addition to the model analyte human IgG (Table 5), this immunosensor configuration was applied to the quantitation of various meaningful biomarkers, namely ALCAM (cancer) [164], CRP (inflammation) [151], and cTnI (myocardial infarct) [144]. In the case of CRP, the use of scFv anti-CRP (30 kDa) as a bioreceptor instead of whole IgG (150 kDa) significantly improved the biosensor response [151]. The mode of grafting of antibody to the AuNR had a significant effect on the performances of the resulting biosensor [148]. Covalent grafting of thiolated anti-human IgG antibody afforded a more sensitive nanoimmunoprobe than electrostatic adsorption to CTAB-capped AuNR sequentially treated with PSS and PAH. In the former configuration, the antibody is preferentially located at the tips of the nanorods where the electric field enhancement is the highest and is comparatively closer to the gold surface. In the latter configuration, the presence of the thick multilayer polymer creates a larger distance between the bioreceptor and the nanorod surface with uniform distribution all over the rods.

### 5.3. Aptasensors

Aptasensors are the class of biosensors using an aptamer as bioreceptor [198]. Aptamers are short synthetic nucleic acid (DNA, RNA) or peptide sequences binding to a specific target with high affinity (comparable to that of antibodies). In addition to their target versatility (ranging from small molecules or ions to whole bacteria and viruses), aptamers are particularly well suited as biorecognition elements of LSPR biosensors owing to their small size enabling the binding events to occur at close range of the gold surface. Moreover, binding of small molecule targets to aptamers usually induces a dramatic change of their conformation (structural switch), which may result in a change of the refractive index at the vicinity of the AuNR and in turn convert into a detectable shift of its LSPR band. Such a direct transduction scheme is hardly achievable with antibody bioreceptors that require a competitive format to enable small molecule detection. Table 6 gathers selected AuNRs-based aptasensors.

A solid-phase aptasensor was built up for the detection of ochratoxin A (OTA), a mycotoxin produced by some *Aspergillus* species and a potential contaminant of foodstuff. Its principle relies on a change of aptamer folding from random and coiled conformation to G-quadruplex structure upon binding of OTA. This and the higher refractive index of OTA induce a red shift of the l-LSPR band proportional to the concentration of analyte in the nanomolar range [199]. Higher sensitivity and wider dynamic range were achieved by co-immobilization of thiol-terminated OTA aptamer and T_3_ onto the AuNR and addition of the G-quadruplex binder berberin for signal enhancement [139]. The same group developed another OTA biosensor with AuNR immobilized on an optical fiber using the same surface chemistry combined with an extremely high RI sensitivity (601 nm/RIU) leading to an LoD of 12 pM [137]. An ATP biosensor was developed by the same group following a slightly different design using AuNR coated with a mixture of T_3_ and split ATP-specific aptamer and a DNA oligomer comprising the other half of the ATP aptamer flanked on both sides by TAMRA fluorophores. Owing to its ability to absorb the visible light, the TAMRA chromophore can strongly couple with the LSPR of nanoparticles to produce large plasmon band shift [195]. Addition of the target triggered the hybridization of the two DNA strands, bringing the two TAMRA entities close to the gold surface resulting in a large shift of the LSPR band. This configuration noticeably enhanced the sensor response in comparison to the simple immobilization of the full ATP aptamer on the AuNR [140].

Another group set up a different biosensor configuration using two AuNR nanoprobes coated by short DNA sequences and the OTA specific DNA aptamer flanked on both sides by sequences complementary to the nanoprobes. In the absence of target, the three of them form sandwiches leading to nanoparticle aggregation. In the presence of target, dissociation occurred because of preferential binding of OTA to its aptamer that in turn led to blue shift and intensity increase of the l-LSPR band [183].

The release of the apoptosis biomarker cytochrome C upon exposure of cancer cells to the chemotherapeutic agent phenylarsine oxide was measured using a biosensor comprising AuNR coated with specific aptamer as transducer and MNP-Ab for target capture. In a microfluidic cell, the target is successively flown over the MNP-Ab while applying a magnetic field. With the magnetic field off, injection of aptamer@AuNR gave rise to the formation of aggregates resulting in the change of the UV-vis spectrum [163]. An LSPR biosensor was set up to detect the presence of the biomarker mucin-1 at the membrane of certain cancer cells using AuNR coated with a specific aptamer and direct reading by UV-vis spectroscopy [136]. An AuNR-based biosensor was designed to assay the cardiac biomarker cTnI with high sensitivity [144]. It combines a short and highly affine peptide aptamer as bioreceptor and filter paper as a solid support. The short size of the peptide recognition element with respect to the more usual antibody enabled to improve the biosensor LoD by one order of magnitude. The l-LSPR band shift undergoes a distance-dependent decay up to 20 nm away from the gold surface as determined by an LbL experiment. This, and the probably higher density of peptide bioreceptors at the surface of the nanorods, explains why the peptide-based biosensor was more sensitive than the corresponding immunosensor configuration.

### 5.4. AuNR-Based LSPR Biosensors Using Uncommon Receptors

Although antibodies and aptamers are the most commonly used bioreceptors for AuNR-LSPR biosensors, several less common biomolecules were also employed as summarized in Table 7. AuNR-based biosensors using sugar bioreceptors were developed to assay food allergens [162] or the cancer biomarker galectin-1 [145]. AuNR-based genosensors were reported for the detection of pathogens [141,143]. A peptide nucleic acid (PNA) biosensor was designed for the detection of circulating tumor DNA (ctDNA) point mutation on the KRAS gene in relation to various cancers, including pancreatic cancer [200]. The sequence of the capture PNA probe was carefully designed to preferentially bind to the mutant sequence and less to the wild-type one. Folic acid-coated AuNR were synthesized and shown to bind to HeLa cancer cells overexpressing the folate receptor [172].

### 5.5. Single Molecule Plasmonic Biosensors

Since the first reports of the use of single plasmonic nanoparticles as independent unit for biosensing [201,202], considerable progress has been made in this direction in the last decade, especially using AuNR as shown in Table 8. The measurements are often achieved by combining a dark field microscope coupled to a microspectroscopy system. The advantages of these single sensors are unlimited especially regarding sensitivity (close to a single molecule) and miniaturization as a minute amount of analyte are needed and very small coverage of nanoparticles required; interparticle distance is important for dark-field microscope imaging. This format is extremely well suited to AuNRs as their LSPR band is often broader than spherical nanoparticles, and, as widely discussed in this manuscript, this band is very sensitive to the AR, which distribution has been largely improved and mastered during the last years, but nevertheless still suffers from variability. Thus, imaging a single AuNR widens the perspectives in terms of sensitivity while narrowing the noise caused by the unequal distribution of the colloids.

Table 8 summarizes the strategies, analytes, bioreceptors, and analytical performances for single molecule plasmonic biosensors. The applications range from the biosensing of model systems such as Biotin/Streptavidin reaching an LoD as low as 1 nM [203] to relevant bioanalytical targets with LoD down to the aM for PSA detection by an aptamer [142].

Miniaturization is one of the main advantages of single biomolecule biosensing that often include a microfluidic device, sometimes allowing for multiplex detection [186]. We expect an increasing attention to these systems with the 3D printing progress.

## 6. Conclusions and Perspectives

All throughout this manuscript, we have reviewed the recent findings related to AuNRs for LSPR biosensing from their synthesis and coating by silica, to their characterization and further surface functionalization, and up to their bioanalytical applications. AuNR synthesis has been extensively investigated over the last decades; therefore, in the first part of the manuscript, we relied on the existing reviews and updated with the most recent literature on the topic. Then, in a second part of the synthesis section, we discussed the methods applied for AuNRs’ coating by a layer of silica sufficiently thin for their potential use as LSPR-biosensors. Although we intentionally highlighted in this section the problems encountered in terms of reproducibility, upscaling, and stability; our main message is that both AuNRs synthesis and their subsequent coating by silica have reached a level of maturity allowing for their extensive use in LSPR biosensing. In the second section of this review, we summarized the experimental techniques allowing for AuNRs characterization at both the microscopic and macroscopic levels. The shape and size, together with the surface charge and the gold content, are measurable data, sometimes applicable for silica shell characterization when needed. In the third part, we comprehensively covered the strategies and methods applied for AuNRs’ functionalization to attach the bioreceptors for their further use as LSPR biosensors. We intended in this part to provide the reader with guidance in choosing the adapted protocol to successively attach the bioreceptor and achieve the desired AuNRs bioconjugate. In the last part, we surveyed the applications of AuNRs in relation to LSPR biosensing classified according to the biorecognition element. The superior optical properties of AuNRs make them unmatchable in terms of efficiency and sensitivity for LSPR biosensing. Their analytical performances are extremely competitive regardless of the used bioreceptor. However, it is important to note that most of the reviewed applications related to LSPR biosensing, with the exception of two, did not include silica coating even if, in the mentioned exceptions, it was demonstrated that the silica shell further improved the overall efficiency of the biosensor. The progress made in the synthesis of AuNR@SiO_2_ highlighted herein promises a booming expansion in their use for LSPR biosensing.

## Figures and Tables

**Table 1:** Summary of the reaction conditions for silica coating on AuNRs using a surface primer exchanged with CTAB and respective silica shell aspects.

Method	AR	Solvent	[CTAB]	Primer	Silica Source	Reaction Time	Shell Thickness (nm)	Shell Feature
Obare et al. [67]	13.0	Water	Minimized	MPTMS	Na_2_SiO_3_	24 h	8	Thin
Perez-Juste et al. [68]	1.9–3.8	Water	Minimized	MPTMS	Na_2_SiO_3_	2 d	5–7	Thin
Li et al. [70]	3.0	Water	Minimized	APTMS	Na_2_SiO_3_	1–2 d	4	Thin
Li et al. [69]	NC	Water	Minimized	MPTMS	Na_2_SiO_3_	2–6 d	0.5–3.5	Ultrathin
Fernández-López et al. [71]	3.5–4.8	EtOH/Water	Without	PEG-SH	TEOS	2 h	4–31	Thin/Thick and dense
Wang et al. [72]	3.2	EtOH	Without	PEG-SH	TEOS	3 h	nc	Thick and mesoporous

**Table 2:** Summary of the reaction conditions for silica coating on AuNRs using a surface primer with respective silica shell aspect. In both cases, the silica source is tetraethyl orthosilicate (TEOS) with no CTAB.

Method	AR	Solvent	Primer	Reaction Time	Shell Thickness (nm)	Shell Feature
Pastoriza-Santos et al. [75]	4.0	2-propanol/Water	PSS, PADH, PVP	2 h	15–40	Thick and dense
Nallathamby et al. [77]	3.6	EtOH	PVP	24 h	2–3	Thin

**Table 3:** Summary of the reaction conditions for direct silica coating on AuNRs with respective silica shell size and aspect.

Method	AR	Solvent	[CTAB] (mM)	Precursor	Reaction Time	Shell Thickness (nm)	Shell Feature
Gorelikov et al. [78]	3.5	MeOH/Water	Minimized	TEOS	2 d	15–60	Thick and mesoporous
Wu et al. [81]	3.6	EtOH/Water	0.1	TEOS	20 h	3–20	Thin/Thick and Mesoporous
Cong et al. [79]	3.0	Isopropanol/Water	0.2	TEOS	20 h	60–150	Thick and dense
Liu et al. [80]	4.3	EtOH/Water	Minimized	TEOS	20 h	10–40	Thick and mesoporous
Abadeer et al. [82]	1.1	MeOH/Water	0.4–1.2	TEOS	20 h	11–26	Thick and mesoporous
Yoon et al. [83]	NC	MeOH/Water	0.4–50	TEOS	24 h	8–21	Thick and mesoporous
Rowe et al. [84]	4.0	MeOH/Water	1.7	TEOS	20 h	NC	Uniform to Dumbbell
Wang et al. [85]	3.9	EtOH/Water	1–9	TEOS	12 h	13–20	Uniform to Dumbbell

**Table 4:** Strategies, analytes, and analytical performances for solution-phase based immunosensors.

Analyte	Format	Analytical Performances	Ref.
hIgG	Direct; aggregation	LoD = 60 ng/mL (0.4 nM)	[130]
Goat anti-hIgG	direct	LoD = 0.4 nM DR = 0.4–100 nM	[156]
HBsAg	Direct	LoD = 0.01 IU/mL DR = 0.01–1 IU/mL	[128]
cTnI	Direct; aggregation	LoD = 10 ng/mL DR = 1–200 ng/mL	[131]
	Sandwich with MNP-Ab	LoD = 1 ng/mL DR = 1–20 ng/mL	[168]
	Direct	LoD = 1 ng/mL DR = 1–20 ng/mL	[132]
CRP	Direct	LoD = 6.2 nM DR = 10–100 nM	[188]
*E. coli O157:H7*	Direct	LoD = 10 CFU Linear response to 5 × 10^4^ CFU	[135]
MC-LR	Competitive disassembly	LoD = 0.03 or 0.6 ng/mL DR = 0.05–1 ng/mL or 1–100 ng/mL	[176]
Aflatoxin B1	Competitive disassembly	LoD = 0.16 ng/mL DR = 0.5–20 ng/mL	[157]
Gentamicin	Competitive disassembly	LoD = 0.05 ng/mL DR = 0.1–20 ng/mL	[187]
*E. coli O157:H7* *S. typhimurium*	Direct; aggregation; multiplex	DR = 10–10^8^ CFU/mL	[173]
Mb cTnI	Direct; multiplex	DR (Mb) = 25–250 ng/mL DR (cTnI) = 1–10 ng/mL	[169]

LoD = limit of detection; DR = detection range.

**Table 5:** Strategies, analytes, and analytical performances for solid-phase-based immunosensors.

Analyte	Format	Analytical Performances	Ref.
Human IgG	Direct	LoD = 61 pM DR = 33–233 nM	[133]
	Direct	DR = 10–40 nM	[146,148]
	Direct; visual detection	LoD = 1 ng/mL DR = 1–10 ng/mL	[175]
ALCAM	Direct	LoD = 15 pM DR = 0.05–30 nM	[164]
CRP	Direct	DR = 1–10 ng/mL	[151]
cTnI	Direct *	LoD = 353 pg/mL	[144]

* solid phase = filter paper.

**Table 6:** Strategies, analytes, and analytical performances for aptasensors.

Analyte	Format	Analytical Performances	Ref.
Ochratoxin A (OTA)	Direct; glass slide	LoD = 1 nM DR = 0.1 nM–10 µM	[199]
	Direct; optical fiber	LoD = 12 pM DR = 10 pM–100 nM	[137]
	Direct; glass slide	DR = 10 pM–10 µM LoD = 0.56 pM	[139]
	Solution; competitive disassembly	LoD = 0.54 nM DR = 1.2–25 nM	[183]
Aflatoxin B1	Direct; glass slide	DR = 10 pM–10 µM LoD = 0.63 pM	[139]
ATP	Direct; glass slide	DR = 10 pM–10 µM LoD = 0.87 pM	[139]
	Direct; glass slide	DR = 10 pM–10 µM	[140]
MCF-7 cancer cells (mucin-1)	Direct; Cells		[136]
Cytochrome c (apoptosis marker)	Sandwich with MNP-Ab for capture; solution; aggregation	LoD = 0.1 ng/mL	[163]
cTnI	Direct; filter paper	LoD = 35 pg/mL DR = 35 pg/mL–3.5 µg/mL	[144]

**Table 7:** Strategies, analytes, bioreceptors, and analytical performances for uncommon LSPR-biosensors.

Analyte	Bioreceptor	Format	Analytical Performances	Ref.
SAV	biotin	Direct; glass slide	LoD = 94 pM (5 ng/mL) DR = 2–2000 nM	[160]
			LoD = 25 ng/mL DR = 25–4000 ng/mL	[171]
			DR = 10–100 nM	[181]
Concanavalin A Peanut agglutinin	4-aminophenyl α-D-mannopyranoside 4-aminophenyl b-D-galactopyranoside	Solution; aggregation		[162]
Galectin-1	lactose	Solution; aggregation	DR = 0.1–100 pM LoD = 0.1 pM	[145]
16S rDNA *Serratia marcenscens*	DNA	Sandwich assay; aggregation	DR = 10 pM–10 nM LoD = 5 pM	[141]
ctDNA (KRAS gene mutation)	PNA	Direct	LoD = 2 ng/mL DR = 40–125 ng/mL	[200]
*Chlamydia. trachomatis* DNA	DNA	Sandwich assay; aggregation	DR = 0.25–20 nM	[143]
Folate receptor	Folic acid	Direct	DR = 100–5000 HeLa cells/mL LoD = 10 cells/mL	[172]

**Table 8:** Strategies, analytes, bioreceptors, and analytical performances for single molecule plasmonic biosensors.

Analyte	Bioreceptor	Format	Analytical Performances	Ref.
SAV	Biotin	Direct	LoD = 1 nM	[203]
Thrombin	Aptamer	Direct	LoD = 10 ng/mL (0.28 nM) DR = 10 ng/mL–100 µg/mL	[204]
			LoD = 0.6 ng/mL (17 pM)	[205]
		Sandwich with Ab	LoD = 1.6 pM DR = 1 ng/mL–10 µg/mL	[206]
NGAL	Ab	Direct	LoD = 8.5 ng/mL (340 pM) DR = 10 ng/mL–1 µg/mL	[165]
PSA	Ab	Direct	DR = 0.1 fM–1 nM LoD = 0.11 fM	[207]
			DR = 1 aM–0.1 nM LoD = 1 aM	[166]
PSA Thrombin IgE	Aptamer	Direct or sandwich with Ab; multiplex; 9-spot array	LoD = 1 ng/mL	[208]
Fibronectin SAV Thrombin IgE	Aptamer	Direct; multiplex	LoD (SAV) = 1 nM DR (SAV) = 1–30 nM	[142]
FtsZ	s1ZipA s2ZipA MinC	Direct; multiplex	DR = 0.2–100 µM	[209]

## Abbreviations

AA	L-ascorbic acid
Ab	Antibody
AD	Dehydroascorbic acid
ALCAM	activated leukocyte cell adhesion molecule
APTMS	(3-aminopropyl)trimethoxysilane
AR	aspect ratio
Asc.^-^	radical ascorbate
Asc^-^	L-ascorbate
ATP	Adenosine triphosphate
AuNR	Gold nanorod
AuNR@SiO_2_	silica coated gold nanorod
BDAC	Benzyldimethylhexadecylammonium chloride
CMC	critical micellar concentration
CRP	C-reactive protein
CTAB	cetyltrimethylammonium bromide
CTAC	cetyltrimethylammonium chloride
ctDNA	circulating tumor DNA
cTnI	cardiac troponin I
DA	dehydroascorbic acid
DDLS	depolarised dynamic light scattering
DDT	dodecanethiol
DLS	dynamic light scattering
DNA	Deoxyribonucleic acid
DR	detection range
DTT	Dithiothreitol
EDC	N-(3-Dimethylaminopropyl)-N′-ethylcarbodiimide
FoM	figure of merit
FWHM	full width at half maximum
GBP-SpA	gold-binding polypeptide Staphylococcal Protein A
HBsAg	Hepatitis B surface antigen
hIgG	human Immunoglobulin G
ICP-MS	inductively coupled plasma mass spectroscopy
ICP-OES	inductively coupled plasma optical emission spectrometry
IGEPAL	octylphenoxypolyethoxyethanol
IgG	Immunoglibulin G
KRAS	Kirsten RAt Sarcoma virus
LbL	layer-by-layer
LC-SPDP	N-Succinimidyl-6-(3’-(2-PyridylDithio)-Propionamido)-hexanoate
l-LSPR	longitudinal localized surface plasmon resonance
LoD	Limit of detection
LSPR	localized surface plasmon resonance
MCF-7	Michigan Cancer Foundation-7
MC-LR-OVA	microcystin-LR *ovalbumin*
MHA	6-Mercaptohexanoic acid
MHDA	16-Mercaptohexadecanoic acid
MNP	magnetic nanoparticle
mPEG-SH	(O-[2-(3-mercaptopropionylamino)ethyl] O’-methylpolyethylene glycol
MPTMS	(3-mercaptopropyl)trimethoxysilane
NaOL	Sodium oleate
NGAL	Neutrophil Gelatinase-Associated Lipocalin
NHS	N-Hydroxysuccinimide
NP	Nanoparticle
OTA	Ochratoxin A
PAH	polyallylamine chloride
PEI	Polyethylenimine
PNA	peptide nucleic acid
PSS	polystyrene sulfonate
PVP	Polyvinyl pyrrolidone
PZC	point of zero charge
RI	Refractive index
RNA	Ribonucleic acid
SAM	Self-Assembled Monolayer
SAV	Streptavidin
SAXS	small angle X-ray scattering
scFv	Single-Chain Fragment Variable
SDS	Sodium dodecyl sulfate
SERS	Surface-enhanced Raman spectroscopy
SHE	Standard hydrogen electrode
SPR	Surface Plasmon resonance
ssDNA	single-stranded DNA
TAMRA	5-Carboxytetramethylrhodamine
TEA	triethylamine
TEM	Transmission Electron Microscopy
TEOS	Tetraethyl orthosilicate
t-LSPR	transverse localized surface plasmon resonance
UTSC	ultra-thin silica shell
XANES	X-ray absorption near edge spectroscopy
